# On the key role of aluminium and other heteroatoms during interzeolite conversion synthesis

**DOI:** 10.1039/d1ra02887a

**Published:** 2021-08-02

**Authors:** Julien Devos, Meera A. Shah, Michiel Dusselier

**Affiliations:** Department of Microbial and Molecular Systems, Centre for Sustainable Catalysis and Engineering (CSCE), KU Leuven Celestijnenlaan 200F 3001 Leuven Belgium michiel.dusselier@kuleuven.be www.dusselier-lab.org

## Abstract

Interzeolite conversion, a synthesis technique for several zeolite frameworks, has recently yielded a large amount of high-performing catalytic zeolites. Yet, the mechanisms behind the success of interzeolite conversion remain unknown. Conventionally, small oligomers with structural similarity between the parent and daughter zeolites have been proposed, despite the fact these have never been observed experimentally. Moreover, recent synthesis examples contradict the theory that structural similarity between the parent and daughter zeolites enhances interzeolite conversion. In this perspective it is proposed that heteroatoms, such as aluminium, are key players in the processes that determine the successful conversion of the parent zeolite. The role of Al during parent dissolution, and all consecutive stages of crystallization, are discussed by revising a vast body of literature. By better understanding the role of Al during interzeolite conversions, it is possible to elucidate some generic features and to propose some synthetic guidelines for making advantageous catalytic zeolites. The latter analysis was also expanded to the interconversion of zeotype materials where heteroatoms such as tin are present.

## Abstract 2

The crucial roles of aluminium in driving and controlling interzeolite conversion, a useful catalyst synthesis protocol, are put under scrutiny.

## Graphical abstract

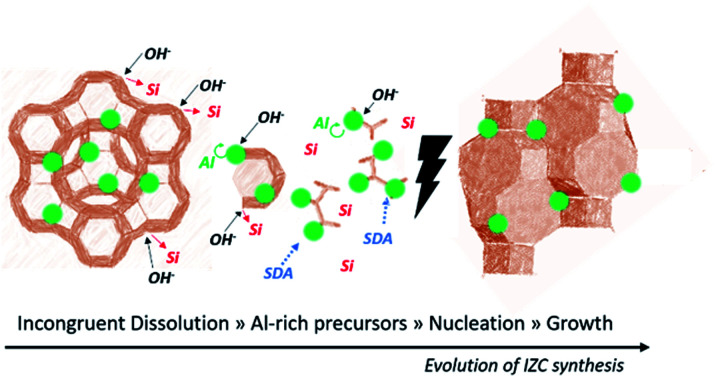

## 1. Introduction

### 1.1 Defining interzeolite conversion (IZC)

**Fig. 1:**
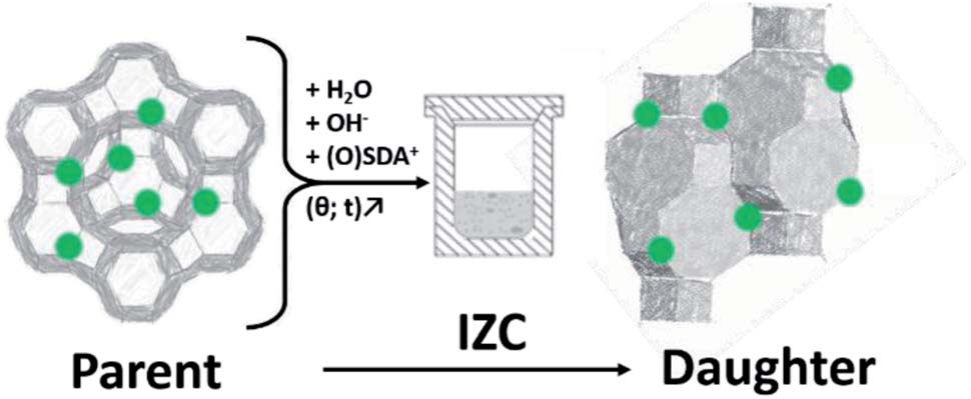
Schematic representation of interzeolite conversion (IZC) from a parent zeolite and some additional reagents (H_2_O, OH^−^ and some SDA). Crystallization of a daughter zeolite occurs at elevated temperature (*θ*) and prolonged exposure time (*t*). Green dots indicate framework Al positions.

Interzeolite conversion (hereafter IZC) is a zeolite synthesis strategy that uses a crystalline zeolitic source (parent) to crystallize another (daughter) zeolite (Fig. 1).^1–3^ Typically this is done in a classic hydrothermal synthesis in batch (opposed to nonconventional *modi operandi*).^3^ Often the term ‘*interzeolite transformation*’ is also used instead of IZC.^4,5^ However, certain polymorphic transformations can also be defined as ‘*interzeolite transformation*’, which may lead to confusion. Examples of the latter are *in situ* recrystallizations of metastable intermediates during prolonged hydrothermal synthesis or after removal of the synthesis liquor (diffusion-less transformations).^6^ These liquor-free operations can be further subdivided in either topotactic^7^ or reconstructive^8^ transformations. An example of the latter is the isochemical GME–AFI transformation at elevated temperatures.^9^ IZC offers unique control over the levers of zeolite kinetics to achieve materials with application potential (Section 2), due to the numerous possibilities to manipulate the liquor (input) and crystallization (conditions).

However, due to the significant number of synthetic levers in IZC (see 3.1), there is a need to distinguish fundamentally different IZC synthesis routes: ‘*true IZC*’ is defined here in cases using solely zeolitic precursors as source for tetrahedral Si and Al oxides (hereafter T-atoms), ‘*partial IZC*’ is used when additional Si or Al sources are used on top of the parent zeolite and ‘*mixed IZC*’ is applied in zeolite recipes combining two or more zeolitic source materials. Furthermore, the IZC analysis is extended towards other microporous framework oxides instead of just silicates and aluminates. The term zeolite in the strict sense, is limited to aluminosilicate versions here, while the broader term (zeotypes) will be used for materials containing heteroatoms other than Al. Interzeotype conversions will be discussed in chapter 4, while drawing parallels to Al and emphasizing future challenges and opportunities in this field.

### 1.2 IZC in perspective

IZC as a synthesis strategy is as old as synthetic zeolite synthesis itself. Synthesis pioneer Richard M. Barrer produced the first zeolite without natural counterpart (KFI)^10^ starting form analcime zeolites (ANA) in geothermal conditions mimicking the Earth's crust, fundamentally an IZC procedure.^11,12^ In the decades that followed, several commercially important synthetic zeolites were discovered in conventional hydrothermal alkaline batch media,^1^ using cheap(er) amorphous sources and diverting the focus away from IZC. A key event was the low Si LTA synthesis discovery in 1956 (ref. 13 and 14) and to date, LTA is still the largest volume scale manufacturing processes of all synthetic zeolites, mainly due to its use in detergents as ion-exchanger (1.4 billion USD in 2018)^15^ or as dessicants.^16^ The first synthetic faujasite (FAU) followed years later,^17^ and was subsequently commercialized for a key oil refining process (fluid catalytic cracking),^18^ an application that represents the largest commercial use of zeolite catalysts by far (∼95% by volume at the start of the 21^st^ century).^19^ Also in the 1960s, the use of organic cations (mainly alkylammonia) as structure directing agents (OSDAs) revolutionized the synthesis field, opening up the possibility to synthesise high Si frameworks with the first patents filed for zeolite materials such as ZSM-5 (MFI)^20^ and zeolite beta (BEA).^21^ The hydrophobic nature and specific shape-selective properties of these materials drove the search for new frameworks in the following decades, initially in the interest of superior oil refining and petrochemistry catalysts,^22,23^ and later also for adsorption (*e.g.* the trapdoor effect)^24^ and other emerging applications such as for clean gas exhausts (NO_*x*_ abatement, SCR).^25^ Furthermore, the synthesis toolbox was expanded to include framework oxides, other than silicates and aluminates alone (zeotypes): borosilicates, stannosilicates, aluminophospates (AlPOs), … A major milestone in this respect is the commercialization of silicoaluminophosphates (SAPO-34) for methanol to olefins (MTO).^26^ Driven by specific applications need in terms of (shape) selectivity, a wide range of frameworks was discovered, exploiting the use of versatile OSDAs as space fillers and often combined with HF as alternative mineralizing agent.^27–29^ To date, more than 250 zeotype frameworks are approved by the international zeolite association (IZA)^30^ and new frameworks are discovered every year.^31^ The size of the pool of potential framework structures is several orders in magnitude larger.^32,33^ More framework discoveries are expected as the synthesis field is taking into account more and more (unconventional) degrees of freedom (DOFs) impacting the (kinetic) route towards new framework materials and compositions.^3^ Among these advanced zeolite synthesis methods, IZC is a promising one. The renewed interest in small pore zeolites, and the promising results of IZC for synthesis of the latter has invigorated the interest in IZC.^1^ IZC as synthesis strategy provides an alternative and often a more extended level of kinetic control, as witnessed from its success to crystallize previously unknown zeolite frameworks such as YFI^34^ and AEI (SSZ-39)^35^ and the expansion of (Si/Al) composition ranges of several others.^1,4^ The power of IZC is best illustrated by its particular success for AEI materials. Despite more than two decades of extensive efforts, the formation of this small-pore zeolite has only been achieved very recently using non-IZC routes.^36,37^ Moreover, these routes are still outperformed by IZC in terms of yield and composition range. Overall, IZC routes offer some additional benefits over conventional routes that may lead to specific, high value applications (see Section 2).

### 1.3 Conventional understanding of IZC synthesis

Since the early 2000s, the Sano group reported numerous contributions of high-performing IZC syntheses.^38^ Without providing (mechanistic) details, a lot of their IZC studies presume a thermodynamic driving force from low framework densities to high framework densities and a kinetically thriving path *via* ‘nanoparts’ with parent–daughter structural similarity.^3,39^ This has indeed been proven to be a very useful guideline for new successful IZC syntheses. However, recent efforts have demonstrated that these simple criteria are inadequate for generalization.^3,5,40,41^ A first remark is given on the thermodynamics. Dense frameworks are indeed more stable than low density frameworks in the case of defect-free silicates, as backed by computational studies.^42^ However, the role of alumina is not accounted for and it is well-known that alumina and its position can significantly determine the relative stability of zeolites.^43,44^ Preferred bonding angles (strain), and thus also the ring sizes (*x*MR) and precise lattice geometries, are most likely at the origin of optimal energetics at particular Si/Al ratios and positions. Additionally, the energetics of the occluded (O)SDA further complicates the thermodynamic picture. Energetic calculations taking into account Al distributions at a relevant (unit cell) scale, as well as solvation effects are currently scarce.^44,45^ The thermodynamics and growth kinetics are difficult to grasp experimentally, even using the most advanced characterization methods of today.^4^ Composite building units (CBU), secondary building units (SBUs) or ring building units (RBU) are all structural (theoretical) classifications of putative ‘nanoparts’, rather than real existing chemical entities. Later, we discuss that any existing theory for kinetically aiding ‘nanoparts’ lacks sound experimental evidence (Section 3.2.3). Suhendar *et al.* indeed concluded that the most suitable predictor for successful IZC are the smallest components, namely the ring building units (RBU).^40^ This is in line with the computational work of Schwalbe-Koda *et al.*, who reported that 65% of the investigated parent–daughter interconversions do not have any CBUs.^46^ Suhendar *et al.* also described the different relation of the proposed RBU intermediate species (4MR, 5MR and 6MR, *i.e.* 4,5,6-membered rings) with respect to Al.^40^ 5MRs are believed to be limited to high Si/Al ratios, as the Löwenstein rule excludes the presence of two Al within one 5MR, whilst the even number RBUs (4MR and 6MR) are more likely to contain Al. This may be a key element in IZC phase selectivity, given the Al densification occurring during incongruent dissolution in the parent (3.1.). Interconversion *via* such Al-rich intermediates may predict the particular success of IZC for 6MR based frameworks (*e.g.* AEI or CHA with TEA as OSDA) and its limited success for particular high silica pentasil recipes (slower ZSM-5 synthesis *via* IZC than *via* conventional methods is reported^47^). In our opinion, a better mechanistic understanding of kinetic control during IZC is needed to develop rational synthesis procedures. However, the required studies on kinetic intermediates and particularly those focussing on altering elemental compositions (Si, Al, SDA contents) have historically been lacking.

### 1.4 The role of heteroatoms (Al) during IZC: scope of the review

The mechanistic understanding of zeolite synthesis is a difficult endeavour. Despite intense efforts, the exact mechanisms leading to specific framework outputs are poorly understood due to the complex nature of crystallizations as a sum of reversible non-covalent (de-)polymerizations^48^ in a heterogeneous environment subjected to temporal and spatial change. On top of this, it is difficult to investigate opaque heterogeneous mixtures *in situ*.^1,4^ IZC provides both its Si and Al in one chemical entity, in contrast with conventional syntheses which typically contain a separate silicate and aluminate source. With amorphous or soluble (non-zeolitic) sources, the order of reactant addition plays a vital role on the heterogeneous synthesis mixtures obtained at elevated temperatures, and thus ultimately the crystal formation,^49^ as changes in this order have been reported to alter the pathways of formation.^50–52^ Such fluctuations related to chemical entities are not expected in single source (true) IZC, thus reducing the complexity of the system. In the majority of IZC cases no gels are formed, which simplifies the mechanistic picture and has aided early IZC investigations such as those of Zones and co-workers.^53–55^ Nonetheless, in-depth mechanistic IZC understanding is lacking.

In line with the recent review of Bruter *et al.*,^56^ the following questions regarding IZC understanding remain unanswered:

- What is the root cause of (kinetic) crystallization differences between zeolites forming from crystalline or amorphous/soluble sources?

- Do ‘nanoparts’ actually participate in crystal growth or during nucleation? And can they be observed experimentally?

- During which stages does Al plays a major role in IZC?

In this perspective, it is proposed that heteroatoms such as aluminium are key players in the processes that determine IZC, providing some answers to these questions and uncovering some generic features of IZC.

This perspective starts by outlining the relevance of IZC (Section 2). Then, an overview will be given on the different processes occurring during all stages of IZC, based on a broad selection of relevant literature data (non-exhaustive) and clues on the role of Al therein (Section 3). The latter analysis is extended to include other heteroatoms such as Sn (Section 4), leading up to a general overview of the field with a summary of key insights and future opportunities (Section 5).

## 2. Advantages of IZC – a rationale

Despite the increasing abilities in zeolite synthesis regarding framework selectivity and synthesis performance,^57,58^ only a dozen zeolite frameworks are exploited commercially.^19,59^ In fact, the vast majority of all commercially used zeolite catalysts are still based on a single framework (FAU), largely due to the economic production process and the high adaptability of the material *via* post-synthesis treatments (*e.g.* steaming of US-Y zeolites). However, post-synthetic treatments encompass a lot of unit operations and modifications which inherently influence more than just the desired parameter leading to suboptimal materials. For example, mesopore formation inherently comes with acid site destruction.^60^ Tailor made synthesis solutions are gaining more traction, especially since the commercialization of Cu-SSZ-13 (CHA). It is believed that tighter market requirements, government regulations and the renewable C-economy (carbon capture utilization, biomass processes) will lead to the further development of speciality zeolites for such high-value applications.^23^

In this respect, IZC is a promising method that could meet the steep requirements for commercialization of high-value applications, potentially benefiting from both superior materials in terms of unprecedented synthesis conditions and physicochemical properties, requirements that are necessary to justify starting from slightly more expensive, but highly pure-crystalline source materials. US-Y (FCC) catalysts sell at 2–4 USD per kg, while speciality (high Si) catalyst sell in the 20 USD per kg range.^61^ Overall, Si and Al sources are not the highest cost in zeolite making. OSDA or very long batch times are regarded as more costly.^62^

In the category of synthesis (process) parameters, IZC typically benefits from shorter syntheses times,^2^ high yields, and the use of alternative OSDAs.^62^ An interesting example in this respect is the potential to replace the expensive tetramethyladamantyl-ammonium (TMAda) used for SSZ-13 (CHA) with the more common tetraethylammonium (TEA) under specific IZC conditions.^63^ IZC often offers multiple advantageous properties in the output materials as well. For one, IZC sometimes opens the ability to selectively make a framework or reach compositions that can not be attained otherwise, as witnessed from AEI investigations (*vide supra*). Secondly, the fast kinetics of IZC generate interesting textural properties. In general, nanosized small crystals are achieved and specific synthesis efforts can lead to mesoporous materials, without the need for helper reagents (*e.g.* a mesoporogen) or post-treatments (bottom-up approaches), for example the synthesis of hollow BEA using a single-step IZC procedure.^64^ A third advantage is the ability to synthesize materials with tailored Al distributions *via* IZC. The latter is relevant for catalysis, especially for redox (multivalent cation exchanged) zeolites.^65^ Most of the IZC benefits summed above are not only related to cost-effectiveness and material properties, but also adhere to principles of green chemistry and safety.^66,67^ IZC can be combined with other promising methods such as solvent-free (solid–solid) synthesis,^68–71^ and seeding methods.^5^ Synergetic strategies can achieve even more promising results. A recent example here is the use of spent (coke-containing) zeolites as effective IZC source yielding hierarchical CHA zeolites.^72^

## 3. Role of Al during the stages of IZC

**Fig. 2:**
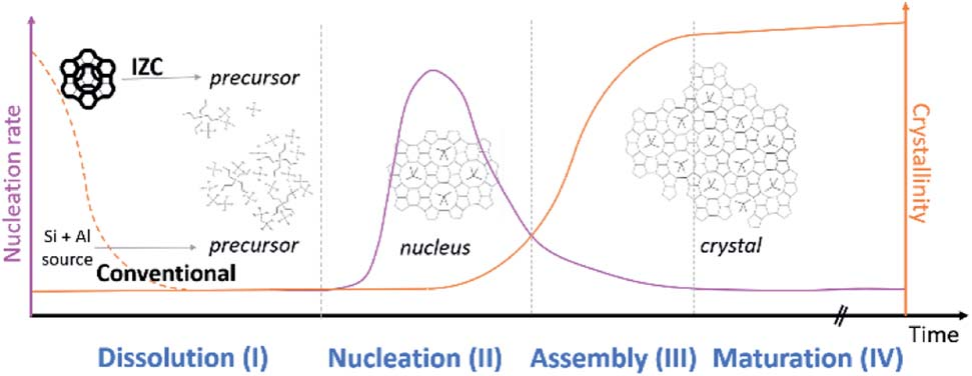
Typical evolution of zeolite crystallization subdivided in four stages. The preselected sources have a fundamental role in consecutive reactions.

The role of Al during IZC synthesis will be evaluated and analysed following the sequence of stages: dissolution (3.1, stage I), nucleation (3.2, stage II), assembly (3.3, stage III) and maturation (3.4, stage IV). These four stages (I–IV) are depicted in Fig. 2. A mineralizing agent (usually OH^−^) will (partially) depolymerize the solid precursors zeolitic in the case of IZC. Dissolution (stage I) will provide the necessary precursors species for nucleation (stage II). During the latter period, also known as induction, nuclei are formed, selective to a specific framework outcome. In the assembly stage (III), the crystal growth occurs autocatalytically, followed by a maturation stage (IV), in which a new equilibrium is found between the formed crystal and its liquid surrounding. This equilibrium formation may also lead to a new crystallization process (Ostwald's rule of stages) as zeolites are metastable (to α-quartz in the case of pure silicates).^44^ It can be expected that silica and alumina (or heteroatoms) will behave differently throughout all stages. This often neglected fact will be the main point of attention throughout this perspective.

### 3.1 Role of Al in (zeolite) dissolution

**Fig. 3:**
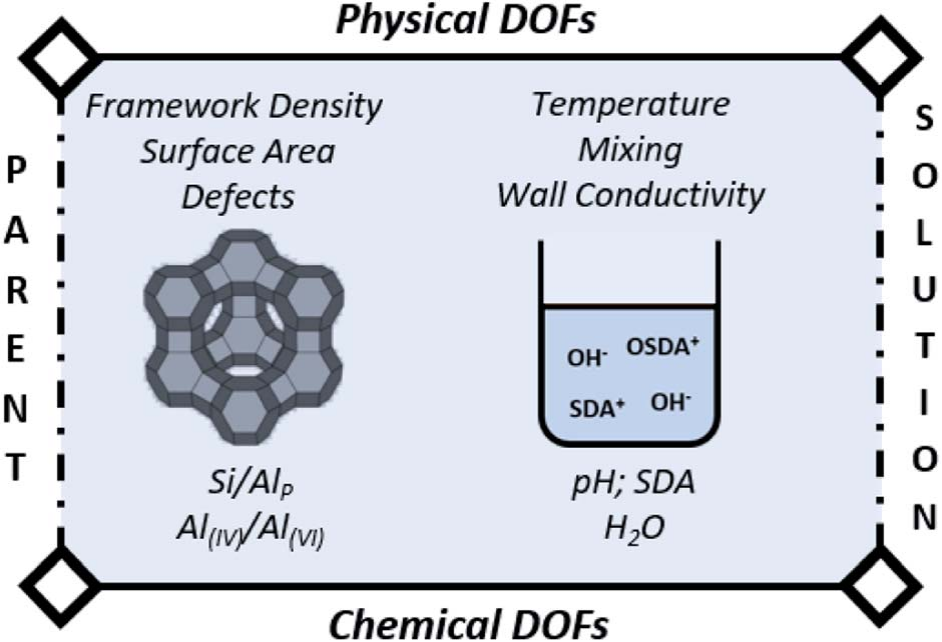
Degrees of freedom (DOF) of IZC synthesis affecting zeolite dissolution, as well as further consecutive growth.

The main variable in (conventional) batch synthesis is time.^3^ Hence, all the “intelligence” for crystallization should be contained at the start.^73^ The latter implies that the initial DOFs (Fig. 3) will be determinant for the further evolution of synthesis. As such, the first (dissolution) stage is in theory the easiest process to follow and actively manipulate in the subsequent jungle of concerted condensation–polymerization reactions. Therefore, we pinpoint the most important degrees of freedom (DOFs) that influence IZC dissolution (3.1.1), followed by an overview of IZC papers highlighting dissolution phenomena (3.1.2) and the construction of a (generic) model for dissolution during true IZC (3.1.3).

#### 3.1.1 Parameters influencing parent dissolution (DOFs)

Parent dissolution is determined by its fundamental physical and chemical properties (*e.g.* surface to volume ratio and Si/Al) and by the properties of the dissolving medium, as summarized in Fig. 3. The latter is composed of a solvent (water), a mineralizing agent (OH^−^) and (in)organic SDAs (Fig. 1). Similar as acid leaching procedures (de-alumination) exploiting solubility of Al at low pH,^74^ the steep increasing solubility of Si above pH = 9 (ref. 75) can be exploited to create mesopores (de-silication).^60,76^ Alumina also dissolves in alkaline media (as Al(OH)_4_^−^) and the solubility strongly increases with pH from approximately 0.03 M at pH = 11 to 0.125 M at pH = 12 in aqueous solutions.^77–79^ However, alumina dissolves to a lesser extent than silica in relevant IZC conditions (pH = 9–14).^80^

Different solubilities of Si and Al provoke gradients in dissolution kinetics or incongruent dissolution,^81,82^ except in Si/Al = 1 cases.^83^ When dissolving alumina or deprotonating silicates one consumes OH^−^, which causes a further drop in solution pH, typically from pH around 13–14 to 11–12. This enlarges the differences in silicates and aluminates solubilities. High pH (>13) is required to achieve sufficient dissolution of Al-rich zeolites (Si/Al of the parent, Si/Al_P_ < 5), whereas siliceous mixtures thrive in lower pH regimes (pH 11–13) in IZC syntheses (see 3.1.2). Next to pH dependence, the dissolution rates of aluminosilicates also strongly depend on the concentration of Al in solution. High aqueous Al concentrations prevent dissolution, especially at high silica concentrations.^84^ Al recondensation prefers reacting with larger silicate species (more branched),^85^ and is thus more likely to linger on zeolitic remnants than silicates becoming fully solvated (*e.g.* oligomers). The reversible nature of condensation/polymerization presented here is regarded as crucial in understanding the generic dissolution and nucleation behaviour proposed below. Čizmek and co-workers provide experimental evidence for the alkaline dissolution behaviour of zeolites in a series of paper spanning both high silica and low silica zeolites.^82,83,86–89^ Next to the strong dependence of dissolution on Al concentration, parent zeolite properties such as crystal size, morphology and defect content are also major contributors to dissolution kinetics (Fig. 3).

In general, dissolution rates at high pH are proportional to the amount of dehydroxylated species (SiO^−^) and their accessibility.^90^ In other words, silanol nests and lattice distortions are the most prone to degradation.^89,91,92^ The latter can cause the formation of ‘hollow structures’, as the initial growth centre has the highest defect probability.^91,93^ Furthermore, reversible Al condensation/polymerizations may take place throughout the parent dissolution process. Similar conclusions can also be drawn from desilication studies in alkaline media. Desilication has a comparable chemistry in only slightly different conditions with respect to IZC (generally lower temperatures and higher concentrations in desilication).^60,76^ It should be noted that non-framework Al can be observed at the outer surface after desilication with NaOH.^94^

In terms of conditions (physical DOFs), increased temperatures both kinetically and thermodynamically promote high solubilities at relevant synthesis conditions.^95^ The temperature-induced increase in kinetics are believed to be so large that reactor wall conductivity becomes a limiting factor.^3,65^ The last physical DOF is mixing, which is believed to be a crucial parameter as it is a determinant for the local availability of Si and Al for further (re-)dissolution and in consecutive processes during the formation of (pre-) nuclei and crystallization.^3^

The role of the last key ingredient (SDA) can be extrapolated from investigations on desilication procedures. In these, both inorganic (Na^+^, K^+^, Cs^+^) and organic base solutions (*e.g.* TPA, tetrapropylammonium) are exploited to introduce mesopores by partial (incongruent) dissolution of the parent structure.^60,96^ Using organic bases for desilication, the alkaline dissolution is much slower and less selective to Al (less incongruent) when compared to inorganic counterparts.^97^ This is likely related to the nature of the organic being lower in charge density. Effects such as the stability, steric hindrance of the (O)SDA and solvation are also at play, in particular during transfer from a hydrophilic aqueous phase to the much less hydrophilic environment of zeolitic pores during synthesis.^98^ In contrast to inorganic hydroxides, (larger) organic hydroxides are not always completely dissociated in water, as witnessed from their lower hydration enthalpies.^99^ This, and stronger stabilization forces between OSDAs and (more hydrophobic) zeolite fractions, lead to a higher affinity to the zeolite surface. Hence, surface coverage may inhibit OH^−^ action slowing down dissolution kinetics, especially for silicates.^97^ Furthermore, the nature of the cation also tends to influence re-insertion of Al and influences its acid strength.^100^ Although the same chemistry takes place during (alkaline) zeolite dissolution as in desilication studies, practical IZC examples are currently lacking to confirm the effect of SDA nature on overall dissolution.

#### 3.1.2 Kinetic investigations on IZC dissolution

In many prominent IZC publications the key-role of dissolution for the consecutive processes is recognized (*e.g.* ref. 101). However, the step is almost never investigated in detail, leaving important IZC dissolution phenomena such as incongruent dissolution and Al densification largely unnoticed (see 3.1.3). This may be in part due to the initial focus on very aluminous IZC (Si/Al_P_ ∼ 1, not showing incongruent dissolution^83^). A series of papers by Subotić and co-workers in the 1980s pioneered the field.^102,103^ They monitored transformation of zeolite A (LTA) into hydroxysodalite (SOD) and zeolite P (GIS) by collecting the liquid phases immediately after synthesis to monitor T-atom concentrations and pH. As a result, they saw equal Si and Al concentration profiles in the liquid phase.^104,105^ Nevertheless, studies exploiting IZC using higher Si/Al_P_ have systematically reported the absence of Al in the liquid phase over the years.^54^ However, most reports have provided little systematic information on the course of Al evolution in solid or liquid, until very recently.

Van Tendeloo and co-workers monitored the liquid phase in a series of IZC starting from Al-rich FAU (Si/Al_P_ = 2.6) using alkali hydroxides (1.2 M MOH with M = Na, K, Rb or Cs).^93^ In all these cases, incongruent dissolution was apparent. The concentration of silicates increased rapidly above 100 mg Si per g zeolite within the first hours. Simultaneously, the concentration of Al in solution also increased rapidly to a range between 5–20 mg Al per g zeolite dependent on the alkali type. Al in the liquid gradually disappeared upon the formation of a new (daughter) phase over the course of a few days. However, formation of a new phase remained absent in the most incongruent experiment using sodium (Si/Al in the liquid phase, Si/Al_L_ = ∼40). In this sodium-containing phase, a daughter zeolite (GIS) only started to form after 16 days. Additionally, they discovered that the liquid-to-solid ratio (L/S ratio), a proxy for water concentration, largely influenced the extent of incongruent dissolution. In the sodium containing system (1.2 M NaOH; Si/Al_P_ = 2.6), the Si/Al_L_ varied between 20 and 150 at a L/S ratio of 100 and 17 respectively. Interestingly, the concentration of Al in liquid solution decreased when there was less liquid in which to dissolve (lower L/S ratio). Perhaps this is influenced by the very high concentrations of Si in dense solution, leading to condensation. Alongside true IZC cases, the paper tested the influence of adding additional Si and Al sources (partial IZC) and the authors concluded that the (Si/Al_L_ ratio of) nutrients available in the solution are a key feature determining phase selectivity.^93^ The majority of parents are not dissolved prior to daughter growth in both the Subotić and Van Tendeloo studies on Al-rich parents (Si/Al_P_ = 1 and 2.6, resp.), as observed from XRD. Such behaviour is also found in other low-Si IZC systems. The results of Norby *et al.* (Si/Al_P_ = 1) demonstrate a total crystallinity (parent + daughter) close to 100% in each stage of the transformation, suggesting that only a minor fraction dissolves.^106^

**Fig. 4:**
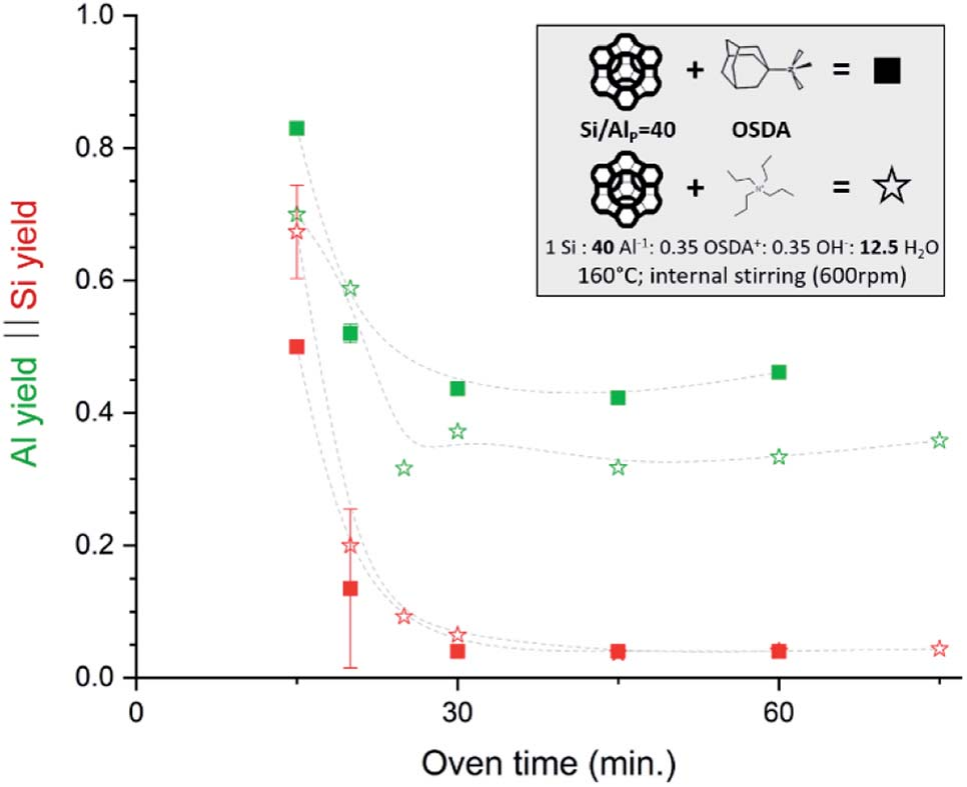
Incongruent dissolution behaviour in true high-Si IZC systems (without alkali cations). This figure has been adapted from ref. 47 with permission from the American chemical society, copyright 2021.

Very recently, some interesting high Si IZC studies have investigated the full course of crystallization, also encompassing the crucial dissolution step.^47,65,107,108^ In 2019, our group published work on FAU-to-CHA (Si/Al_P_ = 40) using simply commercial US-Y (CBV780; Zeolyst) and *N*,*N*,*N*-trimethyl-1-adamantammonium hydroxide (TMAda-OH) at alkali-free molar batch compositions.^65^ Most recently, the investigation was broadened to study FAU-to-MFI under the same conditions, using tetrapropylammonium (TPA) as OSDA.^47^Fig. 4 demonstrates the conditions and the evolution of dissolution in both synthesis systems, in function of oven time.

**Fig. 5:**
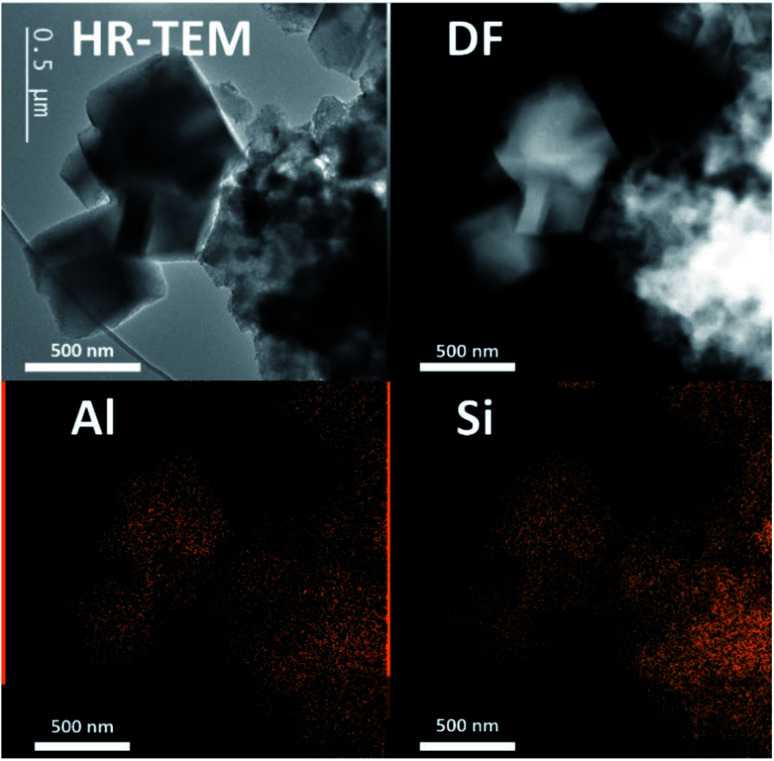
TEM image (left up) and STEM-EDX images highlighting homogenous Si and Al distribution encountered in Al-densified (amorphous) equilibrium compositions collected after 45 min from the synthesis system in ref. 65 (Si/Al_S_ = 3, see Fig. 4).

In both cases, a fast incongruent dissolution is observed within the first 30 minutes, ending at an (apparent) equilibrium composition. The overall yields dropped to 0.04 and 0.05 respectively using TMAda and TPA as OSDA. The same trend is seen for the Si yield in the solid phase (Si yield and solid yield are similar at Si/Al_B_ = 40, see Fig. 4, red). In contrast, the Al yields remained relatively high, around 0.45 and 0.35 for TMAda and TPA (Fig. 4, green) respectively, a clear sign of incongruent dissolution. Furthermore, dissolution seemingly occurs very fast between 15 and 25 minutes. In this period overall yields drop from 0.67 to 0.09 in the TPA case. A slower dissolution pace within the first 15 minutes is due to the thermal resistance of the reactor walls, as dissolution is strongly thermally activated, especially in the absence of alkali metals. This was evidenced from monitoring the liquid phase pH during room temperature aging. After this dissolution period of around 30 minutes, the (overall) composition of the solids remains stable until crystals form (>1 h): Si/Al of the solids (Si/Al_S_) stabilizes at 3 and 4 for the TMAda and the TPA case respectively. Furthermore, both dissolution products are X-ray amorphous, although transmission electron microscopy (TEM) demonstrates the presence of some remnants with the size and morphology of the parent FAU, indicating that these materials are not completely broken down (Fig. 5). Amorphous (gel-like) morphological features were also detected as minor elements. We speculate that gel formation occurs due to the low ionic strength of the liquid environment, which is related to the low content of (dissolved) Al and (Al-attracted) SDA's.

We concluded that reversible condensation polymerization must have taken place to create such Al dense solids, inert for further dissolution. A ‘shielding’ effect of OSDA to prevent further (incongruent) dissolution (as proposed in desilication studies, see 3.1) is not apparent from the examination here. The slight difference in source dissolution and equilibrium distributions (Al yield 0.45 *vs.* 0.35) may stem from subtle differences in OSDA (charge-density) properties (*e.g.* N^+^/C ratio, OSDA geometry and flexibility). The equilibrium liquid–solid composition after initial dissolution and before growth (induction period) creates a unique opportunity to gather in-depth information on the state of the environment in which nucleation occurs in these IZC synthesis, without obscurity from (abundant) gel phases, the influence of other alkali taking part, or the presence of seeds or partial IZC (see 3.1.3).

Another interesting contribution regarding dissolution (and overall) kinetics of IZC is the recent work of Tanigawa *et al.*, although their use of seeds (2% wt of the solids) may impair the analysis.^108^ They converted FAU into CHA using TMAdaOH and NaOH and compared overall growth kinetics at standard batch composition (Si/Al = 20). As sources they used either a low Si/Al_P_ faujasite (Y) parent with an additional Si source, a high Si US-Y parent with an additional Al source, US-Y alone (true IZC), or conventional Si and Al sources (resp. ‘LSY + Si’, ‘HSY + Al’, ‘HSY’ and ‘Si + Al’). HSY + Al, *i.e.* partial IZC from a Si-rich FAU (Si/Al_P_ = 93) and additional Al, produced the fastest dissolution. Interestingly, they measured Si/Al ratios and solid yields of all solid phases. After 3 hours of dissolution, in the case of Si + Al, the solid yield is still at 77% at Si/Al = 13, indicating slow and limited dissolution. In contrast, the solid yield has dropped below 35% for all three IZC cases. In the fastest case (HSY + Al), yield and Si/Al_S_ drop to 13% and 2.9% resp. after two hours. These ratios implicate that the majority (but not all) of Al is present in the solid remnant, while around 85% of Si is dissolved in this short time period, hence demonstrating very incongruent dissolution. The fast kinetics can be mainly attributed to the fast dissolution of a Si-rich zeolite (Si/Al_P_ = 93) and its very defective nature (treated US-Y). In the ‘LSY + Si’ case, dissolution is also fast initially (presumably as the additional Si source is dissolved), but dissolution is limited due to the lower solubility of aluminous Si/Al Y and in absence of fast crystallization (no cascade of reactions). The true IZC case examined here (HSY) encounters slower initial dissolution kinetics than both the partial IZC counterparts, a non-intuitive result. We propose that this may be related to the less defective nature of the parent (*vs.* HSY + Al) and the ‘protective’ effect of incorporated Al in neighbouring Si species, limiting Si dissolution compared to silica dissolution from amorphous sources (LSY + Si). Note that Al-rich conditions are observed in the solid phase of all 3 examined IZC cases just prior to their nucleation, a potential key ingredient for the latter (see 3.2.3).

**Fig. 6:**
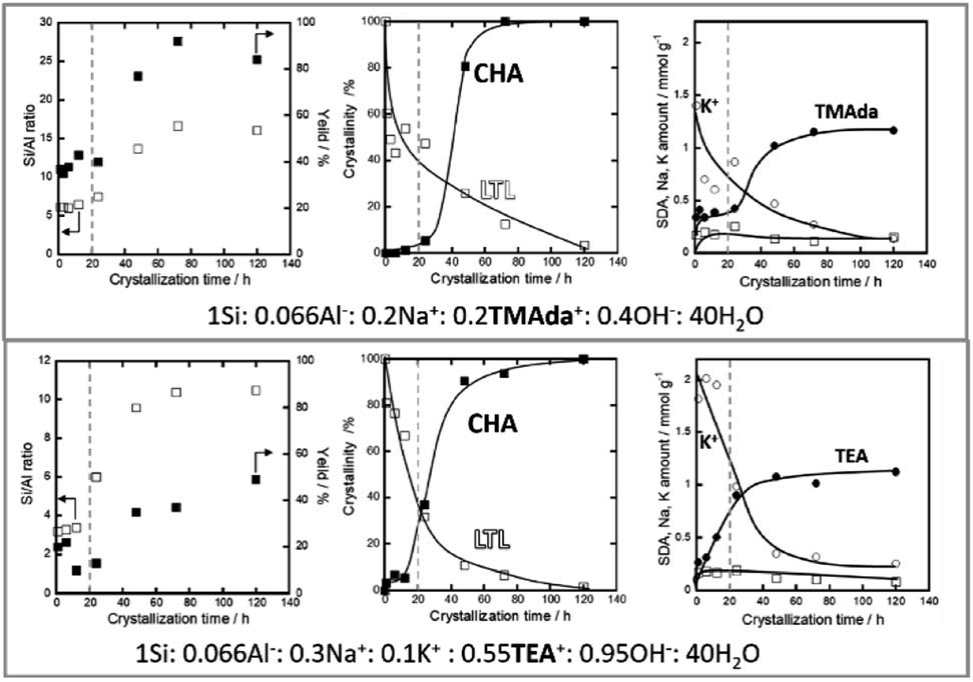
Evolution of LTL-to-CHA partial IZC (Si/Al_P_ = 3.0; Si/Al_B_ = 15), adapted from Nishitoba *et al.*^107^ A period with stable compositions is discerned prior to nucleation and growth (dotted line). The molar compositions do not account for the high K-content in the parent phase (16% K_2_O).

Additionally, Nishitoba *et al.* assessed Si/Al_S_ ratios and solid yields in interesting IZC systems, along with SDA/Al and crystallinity information over the full course of crystallization. However, it must be said that the system is a partial IZC and in the presence of a high quantity of seeds (10 wt%). FAU-to-CHA IZC (Si/Al_P_ = 2.6; Si/Al_B_ = 15) and LTL-to-CHA IZC (Si/Al_P_ = 3; Si/Al_B_ = 15) were thus investigated using TMAda and Na as SDA as well as using a combination of TEA, Na and K (presumably at higher pH).^107^ From these four systems, it was observed that dissolution was faster in the TEA systems (logical from a pH viewpoint) and that it occurred even faster for the FAU case *versus* starting from LTL. Furthermore, the parent effect on dissolution was more pronounced than the pH effect, which highlights the importance of parent properties. They subscribe this effect to a thermodynamical parameter (framework density). However, it is suggested here to assess morphology and (defect) chemistry of the zeolitic parents to validate -or likely reject-this assumption. Nishitoba's LTL source contains a high fraction of potassium (16 wt% K_2_O), which may also be a key determinant in dissolution kinetics. High potassium concentrations are found to persist during the gradual LTL dissolution (Fig. 6, right). In accordance with the other high Si IZC studies, significant incongruent dissolution was observed in all four studied systems, with the most (incongruent) dissolution observed in the more alkaline (TEA) media (Fig. 6, down left, very low Si/Al_S_). The systems with LTL parents experience stable solid yields and Si/Al_S_ ratios over a long period (1–12 h) of oven heating (443 K) until disturbed by nucleation and crystallization (Fig. 6, central). Hence, these solids show similar aluminous sols as those detected at equilibrium compositions by Devos *et al.*^47,65^ Despite the stable composition in terms of yield and Si/Al_B_, the crystallinity of LTL decreased significantly during this (macroscopic) equilibrium period (Fig. 6, central). Moreover, K contents of the solids also decrease, but no other significant trends were found in terms of composition for other SDA molecules (Na; OSDA) in the time period before nucleation (Fig. 6, left). Kinetics of dissolution and growth were too fast to analyse when using the FAU sources, as likely is the case in most studied IZC systems.

#### 3.1.3 Distinct dissolution behaviour in IZC: a mechanistic proposal

**Fig. 7:**
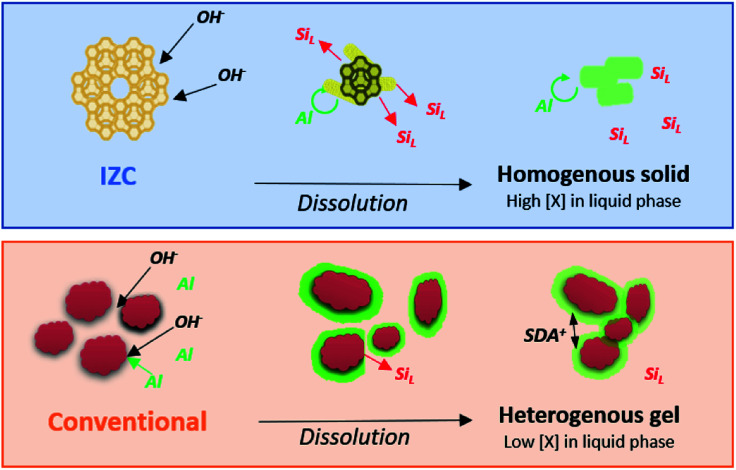
Physical states and Al concentrations (red = siliceous, green = aluminous) through the stages of dissolution starting from high Si/Al_P_ materials. Relatively homogeneous distributions of Al (no gradients) are found within the remnant solid after IZC dissolution as opposed to the conventional case yielding a heterogeneous gel.

IZC has (parent) zeolite dissolution as a starting point, in contrast with synthesis starting from amorphous sources. This yields a significantly different context of physical states and precursor species involved in nucleation, growth and is pivotal to form particular nuclei (phase selectivity). In this section, the particular (physical) outcomes of IZC dissolution are compared to those from other (conventional) systems (see below, Fig. 7). In contrast to the conventional theory involving precursor species that do not require complete dissolution, so called ‘nanoparts’, we approach the problem here by sketching the physicochemical context at the onset of nucleation in IZC mixtures and comparing them to those of conventional (aluminosilicate) zeolite synthesis. Therefore, we first summarize the gathered knowledge on zeolite dissolution:

- The extent of dissolution is mainly determined by pH, temperature, dilution (L/S ratio), SDA interaction and the chemical properties of the zeolite source (Fig. 3).

- Zeolitic sources dissolve incongruently due to the higher reactivity of hydroxides with silicates than aluminates.

- The liquid phase is rich in silicates. The absence of Al prevents aggregation and gel formation.^109–111^

- A solid persists, enriched in Al.

- The remnant solids have the parent morphology and amorphous gel formation is not common.^54,65,101^

- Aluminous parents only dissolve partially before nucleation and growth occur.

- Siliceous parents achieve an ‘equilibrium dissolution phase’ before nucleation.

- At these equilibrium compositions, microscopic changes still occur, such as the depletion of remnant parent long range order (crystallinity).

As such, we propose an incongruent IZC dissolution towards homogeneous and Al rich containing sols in a siliceous liquor with low tendency to form amorphous gels (Fig. 7). If Al is removed from the framework, it should rather breakaway as large aluminosilicate domains than as monomeric Al, because the electronegativity of Al protects their bonds with silicate neighbours. Moreover, monomeric dissolved Al are believed to quickly reassemble within the crystal, far away from other (charged) framework Al, in line with the Löwenstein's^112^ and Dempsey's^113^ rules of charge separation. Hence, a zeolite may dissolve in putative Al-rich ‘*nanoparts*’, however, this does not automatically imply that these are involved in nucleation and assembly (see below).

In contrast to IZC, most conventional systems (aluminosilicate hydrogels) have a dissolution step involving (more) monomeric Al (Al(OH)_4_^−^). We presume this leads to physical states present at the onset of nucleation which have a much more heterogenous spatial distribution of Si and Al, as prompted from the extensive analyses of Ren *et al.*^109,114–116^ Soluble Al present in the early stages of dissolution will preferentially complex with (dehydroxylated) larger silicate species (*vide supra*).^85^ This leads to surface-enrichment of Al on amorphous silicates (*e.g.* colloidal silica or silica sols) and makes those resistant to dissolution. It can be interpreted that dissolved Al(OH)_4_^−^ forms a passivation layer (Fig. 7), coupled with the action of SDA's. These positive charged entities propel aggregation of anionic colloidal particles that contain mainly silicates. Such densification can be beneficial for nucleation but hampers further dissolution and limits the extent of supersaturation and swift growth (see below). Acknowledging the importance of physical states and its heterogeneity in zeolite crystallization kinetics is therefore very important.^49^Fig. 7 summarizes the dissolution behaviour of IZC and conventional sources and their resulting physical states as hypothesized from the key role of dissolved Al. Note that slower dissolution kinetics are often related to better framework selectivity.^36^ We suggest that this may have to do with the particular physical states formed due to the dissolution behaviour of sources (in particular of Al). In this way, (slightly) alternative physical states (and precursor species) can be formed after (more extensive) dissolution than with alternative Al sources, monomeric Si (TEOS), or the application of ‘stepwise’ procedures.^52,117^

Despite the applicability of the (proposed) model for the particular IZC dissolution behaviour, a lot of questions remain:

- Is the proposed model valid for a large range of Si/Al_P_, or rather for high Si values such as in ref. 65.

- What is the role of specific SDAs (*e.g.* SDA-OSDA combinations) on dissolution?

- What is the speciation of the dissolved silicates and aluminates after IZC dissolution? Are they similar to dissolved species from other physical states?

- Does their speciation alter upon nucleation and growth?

Some of these questions are partially answered in the following sections on nucleation, growth and maturation. However, it is safe to say that the physical state present at the onset of nucleation and growth is likely the most determinant factor in the whole IZC process.

### 3.2 Role of Al in nucleation

The number and size of IZC-synthesized crystals indicate that nucleation is a quick process.^65,118^ In the following, the origin of seemingly generically fast nucleation in IZC mixtures is dealt with, considering the context of physical states obtained by dissolution (3.1.3), the nucleation mechanism, the involved chemical species and the role of Al therein. First, a general theory on zeolite kinetics and thermodynamics is presented.

#### 3.2.1 Kinetic and thermodynamic pathways of nucleation

In order to grow specific nuclei, a certain degree of supersaturation in required. The degree of (super)saturation (*S*) quantifies the ratio of actual and equilibrium concentrations (*S* = *X*/X*) of precursor solutes (*X*), hence *S* > 1 for supersaturation.^111^ Nucleation is a thermally activated process and supersaturation increases exponentially with heating and source dissolution. At a critical supersaturation level (*S*_crit._) viable nuclei form. *S*_crit._ can be decreased by heterogeneous nucleation, given a reduction in surface energy. Nucleation rates initially increase exponentially with increasing supersaturation (*S*), but encounter a maximum due to enhanced viscosities (gel formation) which limits diffusion (Fig. 2), and the availability of precursors (*X*).^111^

**Fig. 8:**
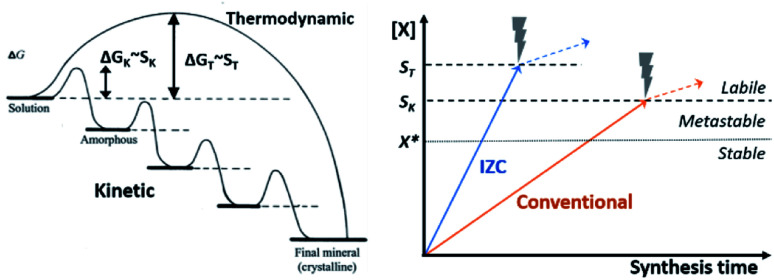
Evolution of zeolite synthesis under thermodynamic (T) and kinetic (K) control (left), as adapted with permission from Cölfen *et al.*^120^ Copyright 2003 Wiley. Whether a one-step route (thermodynamic) to a final crystal phase or the sequential kinetic route is followed depends on the energy of activation (Δ*G*) which is dependent on the degree of supersaturation for each route (*S*_K_ or *S*_T_). The graph on the right depicts precursor solubilities (*X*) in function of synthesis time upon source dissolution (IZC, blue & conventional, orange, *cfr.*Fig. 7). Crystallization occurs above X*, corresponding to equilibrium solubility (dotted line). A significant degree of supersaturation (*S*_crit._, dashed lines) is required to spark nucleation. The thermodynamic pathway requires higher *S* (*S*_T_) than the kinetic pathway (*S*_K_) and the mechanism of nucleation, may influence *S*_crit._. After nucleation (*X* > *S*_crit._, dashed lines), solubilities are influenced by other parameters than just source dissolution (dotted arrow).

Classic nucleation theory predicts a critical nucleus size as tipping point for thermodynamically favoured self-assembly of unit-cells, *i.e.* crystallization.^119^ The classic nucleation theory is valid for single-step addition of small precursor species (<unit cell) and presumes unidirectional growth towards the thermodynamically most stable crystal nucleus.^119,120^ However, crystallization routes are determined by the activation-energy barriers (Δ*G*) of nucleation, growth, and phase transformation of the (potential) phases (Fig. 8, left). Rather than a direct thermodynamically controlled route to the most stable phase, crystallization can also occur through a sequence of intermediate phases with increasing stability (kinetic control).^120^ Activation-energy barriers (Δ*G*) depend on the supersaturation (*S*) of precursor species, as well as activation energies for interconversion and the presence of additives (*e.g.* SDAs). This involves structural and compositional modifications of the amorphous and crystalline intermediates.^120^ Zeolites synthesis is believed to mainly occur *via* kinetic pathways,^5,44,86^ with a large contribution of heterogeneous nucleation.^121^

Kinetically controlled routes allow less severe conditions for growth, hence a lower degree of supersaturation (*S*_crit._ > X*) is required for nucleation. However, kinetic (amorphous) intermediates can slow down the synthesis time of a targeted product that is thermodynamically more stable, as the relative stabilities of the intermediates slows down the formation of the desired product (Fig. 8 left, final mineral). Even worse, phase selectivity can be diverted to undesired phases *via* these intermediates. Ideally, no intermediate (gel) formation occurs during swift crystallization of a targeted zeolite. The later is the case in particular at high *S*. At a certain *S* (*S*_T_), crystallization follows a more direct thermodynamic route than the kinetic route requiring lower supersaturation (*S*_K_), as depicted in Fig. 8 (right, dashed lines).

The above insight on nucleation theory provides some guidelines to actively control the crystallization mechanism by controlling the degree of solute species (*X*), hereby influencing the supersaturation requirements (*S*_crit._) for the direct (*S*_T_) and kinetic (*S*_K_) pathways. It can be interpreted that supersaturation is relatively higher in IZC systems than in (compositionally similar) conventional systems due to the inherently faster dissolution kinetics related to the Al distributions in the dissolving physical states (Fig. 7, see 3.1.3). As such, faster and higher supersaturation is expected during IZC in comparison to conventional source counterparts (Fig. 8, right), as experimentally evidenced from the generally smaller output (daughter) crystals with ‘rough’ morphologies encountered from IZC mixtures,^65,118^ and by the absence of gel-phase intermediates.^54,122^ As such, based on a preliminary analysis of classic nucleation theory and the dissolution behaviours of IZC mixtures, it could be suggested that precursor concentrations are at least as important as the precursor speciation for nucleation and phase selectivity. Despite the higher solute concentrations, allowing a more direct thermodynamic pathway (*S*_T_), IZC may also provide unique opportunities to enable zeolite growth *via* kinetic pathways (at low *S*_crit._, or *S*_K_) which would otherwise not crystallize at all (*e.g.* AEI).

#### 3.2.2 Mechanisms of IZC nucleation

Earlier we discussed the conventional mechanistic understanding of IZC presuming it is driven thermodynamically by framework densification and kinetically by the involvement of putative ‘nanoparts’. It was evidenced that these criteria are not sufficient to explain the mechanism, hence a more in-depth mechanistic understanding is required, especially regarding the role of Al (1.3). Davis *et al.* have reported a strong dependence of crystallization mechanism on the Al content for conventional syntheses,^123^ which seems no different for IZC systems.

Norby *et al.* proposed 5 potential mechanisms of IZC:^106^ (1) internal structural rearrangements (without the involvement of a solution or amorphous phases), (2) a solution-mediated transformation (nucleation in solution), (3) a gel-mediated process (involvement of a gel phase), (4) a surface-mediated transformation (‘non-selective’ heterogeneous nucleation), and (5) structural similarity enhanced (epitaxial) transformation. The last criteria will also be referred to as ‘selective’ heterogeneous nucleation.

The first mechanism (solid rearrangement) was proposed for the FAU-to-ANA IZC (Si/Al_P_ = 6.7).^124^ The authors monitored morphologies *via* TEM. The FAU parent became unstable and ANA is formed from the outside-in, without changes in particle size and morphology. Apart from this solid rearrangement proposal (1), all the others involve a liquid phase and rely on supersaturation criteria for nucleation and growth. In theory, the required supersaturation for nucleation (*S*_crit._) in these proposed mechanisms is believed to be lower for the pathways with higher numbers (*i.e.* from (2) to (5)). On one hand, fast nucleation in IZC systems can thus be a consequence of either high supersaturation (*S*) due to superior dissolution kinetics (3.1.3), or on the other hand, nucleation can be fast due to crystallization modes requiring lower supersaturation (a drop in *S*_crit._). Note that a combination of both is also possible. Apart from the FAU-to-ANA study (mechanism 1), no other IZC materials shows morphologic correlation between the parent structure and daughter structure, which indicates transformation *via* solution.

A lot of early investigations highlighted the importance of solution-mediated nucleation (2), which supports the theory of superior dissolution kinetics in IZC (see 3.1.3), while newer studies frequently ascribe IZC success to structural similarity enhanced mechanisms (5). Subotić *et al.* performed the first extensive mechanistic IZC investigation on LTA to SOD and GIS (Si/Al_P_ = 1, OSDA-free). They observed discrete particles of parent and daughter zeolites throughout the whole process by monitoring the evolution of parent and daughter morphologies and by elemental analysis of solid and liquid, from which they concluded that IZC growth is solution-mediated (2).^102,103^ Such observations of discrete particle growth were also made by Norby *et al.* in their LTA to ABW system (Si/Al_P_ = 1; OSDA-free) *via in situ* powder diffraction and *ex situ* SEM.^106^ However, their level of detail allowed the observation of ABW morphologies located at the most surface-strained locations (faulty crystal edges), indicating surface-induced nucleation (4). Similar observations were made later by Davis and co-workers^125^ and Van Tendeloo *et al.*^93^ No gel-phases were observed in all the above (OSDA-free and seed-free) transformations in aluminous media (Si/Al_P_ < 3), excluding mechanism (3). Nucleation may be surface-mediated whilst the consecutive growth appears to be mainly solution-mediated. The importance of the solution is further backed by solid–liquid separation experiments. The liquid phase allows nucleation of the expected daughter zeolite even after removal of the solid phase with its significantly different elemental composition (Si/Al_L_).^93^

Higher silica IZC (Si/Al > 5) usually involves an OSDA. In these processes, surface-mediated nucleation is the most frequently proposed mechanism. A pioneering effort in this regard is the study of GIS-to-LEV by Zones *et al.*,^101^ although this is a partial IZC and involves a significant amount of added Si. It is proposed that the OSDA serves as a phase-transfer agent, due to the amphoteric nature of most OSDA's.^101,126^ Presumably the OSDAs can provide beneficial charge balancing and solvation energies to stabilize regions with variable Si/Al. The latter is particularly important for high Si/Al IZC given the large extent of incongruent dissolution in these syntheses and the large gradients in Si/Al encountered at the interfaces of both.

Whether heterogeneous nucleation is facilitated by structural similarity between building units (‘nanoparts’) and a parent structure is a matter of ongoing debate (mechanism 4 *vs.* 5). One could convincingly suggest that heterogeneous nucleation occurs unselectively (4), hence independent of the presence of ‘nanoparts’ species with structural similarity, as IZC syntheses with no common SBUs also achieve nucleation much faster than their amorphous counterparts, even in the absence of OSDAs.^127^ A good argument in favour of non-selective heterogeneous nucleation can be found from our kinetic study of FAU-to-MFI and FAU-to-CHA (Si/Al_P_ = 40).^47^ In this work, it was demonstrated that nucleation occurred swiftly around one hour of FAU-to-MFI *vs.* 16 hours for amorphous (non-IZC) counterparts. The same time is required for FAU-to-CHA in equimolar compositions, which is a more structural-similar transformation. Non-selective nucleation may also be suggested from some of the studies of Rimer *et al.*^5^ In their recent contribution of Sr-CHA zeolites using seeds (10 wt%), strictly not an IZC contribution, they found a comparable reduction of synthesis time from 14 days to 3–4 days when using either CHA or FAU seeds.^128^ From the latter it can be interpretated that FAU serves as a non-selective heterogeneous nucleation (4) centre. Using CHA seeds sparked nucleation (only) a little faster, which may reveal a minor contribution of structural similarity enhanced nucleation (5).

**Fig. 9:**
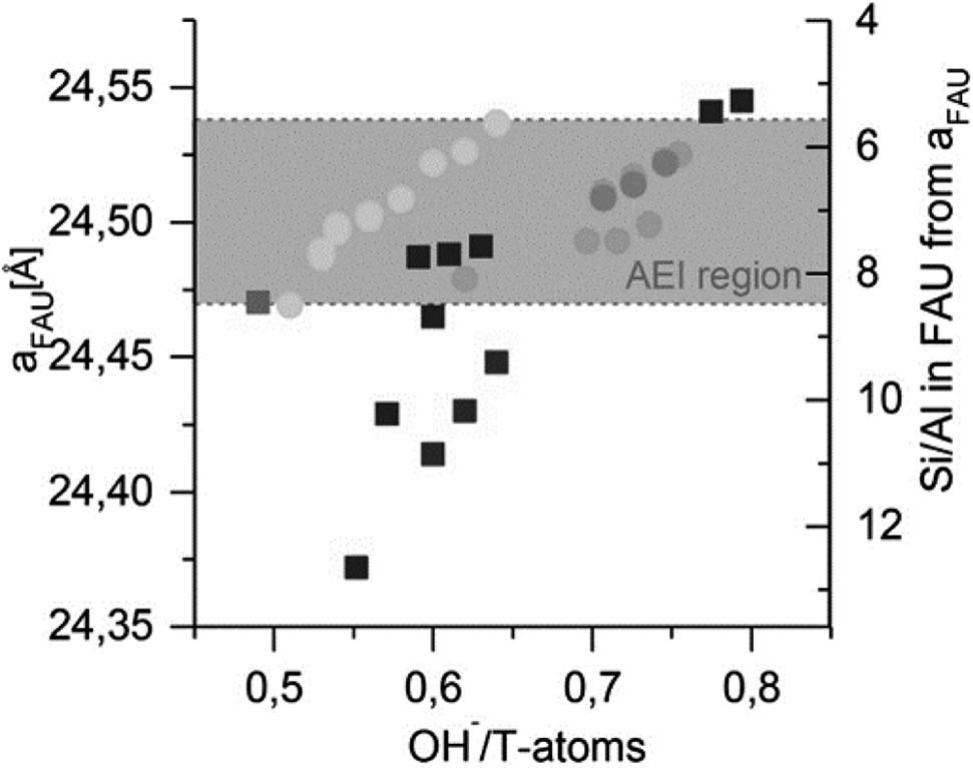
FAU lattice parameter ‘*a*’ after synthesis and corresponding Si/Al in FAU in relation to the initial OH^−^/T-atoms in the gel. Circles indicate FAU + AEI product mixtures, discriminating from products solely containing FAU (squares). AEI reflections are only obtained in the grey region. Grey-scales indicate particular synthesis recipes variations (partial IZC; Si/Al_B_ variations, …). Reprinted with permission from Boruntea *et al.*^132^ Copyright 2019 Elsevier.

In contrast to the suggestions above in favour of non-selective mechanisms (4), Boruntea *et al.* have recently found evidence that could suggest the occurrence of ‘selective’ surface mediated growth (5). In their recent contribution on (difficult to crystallize) FAU-to-AEI and FAU-to-AFX, they found matching lattice parameters of the remnant parent – proportional to the Al content^129^ – as an important prerequisite for successful AEI formation. The authors found a narrow window of OH^−^/T-atoms ratios that produced AEI. As depicted in Fig. 9, too low and too high Si/Al_P_ or pH (OH^−^/T) were found unsuitable for IZC. Deriving the Si/Al from the lattice parameters (XRD) of the remnant FAU,^129^ it was concluded that AEI nucleation could take place in the Si/Al_S_ range of 6–8. Whether matching chemical compositions (Si/Al) or the presence of specific structural units with matching geometry, or both, are important to stimulate structural similarity enhanced nucleation (5) and growth remains uncertain. A more in-depth investigation, taking into account the liquid phases, is necessary to investigate the root cause of AEI success (see outlook section). Either way, the AEI case once more highlights the importance of Al.

The transformations with low selectivity (*e.g.* AEI growth) likely requires fast and extensive dissolution, while this is not necessarily needed for easily crystallizing phases, allowing heterogenous (stable) gels as intermediates and more kinetic pathways (lower *S*_crit._). Other difficult to crystallize systems, such as OSDA-free transformations (*e.g.* ‘green synthesis’ of BEA),^130^ operate at border compositional ranges may heavily rely on the low supersaturation nucleation *via* a structural similarity enhanced mechanism (5) in parallel with the suggestion of Okubo *et al.* in their works on seed-assisted OSDA-free syntheses.^131^ In contrast, Dos Santos *et al.* recently argued that structural similarity should not take an important role in IZC as they could successfully synthesize MFI without an OSDA *via* partial IZC (Si/Al_P_ = 3; Si/Al_B_ = 25; no seeds).^127^ Note that a distinction is made here deliberately between nucleation (phase selectivity) and growth (yield), as supersaturation and growth may significantly alter during the assembly phase (see below).

#### 3.2.3 Chemical species involved in IZC nucleation

From the analysis above, we derived that most IZC nucleation mechanisms involve delivery of nutrients from solution. An increased knowledge on the chemistry of the putative precursor species (*X*) in solution may help to unravel IZC understanding.^133^

Building block descendants formed from the dissolution of amorphous sources may have a different connectivity than those descended from crystalline sources, as yet presumed by Subotić *et al.* (S-species and Q-species).^103^ Regarding the physical states involved in such dissolutions, it was reasoned above that IZC based syntheses have a larger amount of T-atom species in solution, due to a lower content of undissolved Si (shielded) regions (Fig. 6, Section 3.1.3). As such, species with higher connectivity can be expected in such IZC mixtures. However, it is questionable whether fundamentally different growth species (*X*) can be formed from condensation of dissolved amorphous (non-IZC) species *versus* those formed from the dissolution of crystalline zeolitic fractions as the same reversible chemistry takes place. However, the different kinetics and extent of dissolution between IZC and non-IZC routes (see 3.1.3, Fig. 7) at the onset of nucleation suggest the possibility for a difference in species taking part during assembly. At least on the macroscopic scale, such differences have been observed (classic growth *versus* non-classic aggregate growth^134,135^). Dissolved silicate and aluminate entities in alkaline media condense *via* an S_N_2 mechanism requiring a leaving group.^136^ The formation of Si–O–Al bridges requires lower activation energies than Si–O–Si formation.^137,138^ As such, dissolved Al plays a crucial role in building condensed structures.^139^ Computational efforts at relevant synthesis conditions indicate that cyclization of condensation products is thermodynamically favoured and SDAs such as Na^+^ or Ca^2+^ further increase condensation tendency.^138^ Of course, the kinetics of oligomerization should also be considered. Swaddle explains that cyclic oligomers cannot readily expand their coordination number beyond four (required for S_N_2)^137^ and so cyclic oligomers are less reactive than their acyclic peers. Furthermore, according to Swaddle, zeolite assembly is more likely to take place *via* the addition of small acyclic oligomers rather than from (unreactive) cyclic components.^137^ As a consequence, there may not be any advantage of using pre-existing structures (CBU, SBU, *etc.*) in solution based-growth. This was also augmented by Knight in 1990 as no correlation could be found between the species detected in solution and the type of zeolite crystallized therefrom.^140^ Furthermore, Cundy and Cox argument that only simple species propagate networks, as larger species have a larger probability for bad docking positions, and are subsequentially redissolved.^86^ In this regard, they also proposed that highly symmetric oligomeric species may have a higher probability for correct assembly, which leaves a door open for the involvement of pre-existing structures (not entirely dissolved) in IZC or seeded syntheses.

Recently, attempts have been made to trace IZC intermediates in time using Raman spectroscopy.^141–143^ Unfortunately, the cumulative relative crystallinities of parent and daughter zeolites remains relatively close to 100% in these works. In such cases, with no more than 4% of T-atoms in solution,^141^ it is virtually impossible to correlate pre-existing species (*X*) to growth (see 3.3) as one is not able to distinguish structural elements present within crystalline parent or daughter zeolites from the contribution of independent growth units.^56^ The same experimental set-up should also be performed at synthesis conditions with at least 50% of the T-atoms dissolved (*i.e.* at higher Si/Al) for clear experimental observation of potential structural growth units. The later was somewhat addressed in an interesting contribution of the Sano group using FTIR back in 2008.^144^ In FAU-to-BEA (Si/Al_P_ = 23; alkali-free), they encountered an X-ray amorphous intermediate phase (between 2 h and 24 h). FTIR in these regions did not reveal any FTIR visible rings. At first sight, one would interpret this as a case against locally organized aluminosilicate rings. The authors rightly point out that such detailed structure cannot be proven. Instead, they applied a consecutive hydrothermal treatment with a mesoporogen added to the synthesis mixture of the amorphous intermediate phases (at 2 h and 18 h) and analysed the output of hydrothermal synthesis after 5 days. Based on the presumed stabilising role of mesoporogens on zeolitic precursors, seeds and/or fragments, they deduce that the amorphous intermediates should have contained pre-existing ordered aluminosilicate species larger than 6-MRs.^144^ Note that this is only very indirect experimental evidence for the (since then) prevalent theory of ‘nanoparts’ involved in successful IZC.^39^ Recently, the group has given more experimental evidence for the presence of specific aluminosilicate oligomers in IZC solutions *via* (*ex situ*) electrospray-ionization mass spectrometry (ESI-MS).^36,108^ Higher *m*/*z* intensities (*m*/*z* range 300–1000) were found after prolonged synthesis, indicating condensation and adduct formation (with water and SDAs). However, the numerous potential oligomers (and SDA adducts) impede detailed assignment of the output *m*/*z* ratios, let alone provide information of their involvement in nucleation and growth.

Overall, the current state of the art investigations on IZC mechanisms are insufficient to confirm ‘nanoparts’, let alone capture their role in IZC, in line with other IZC review papers.^4,56^ It would be interesting to experimentally detect the role of Al in oligomerization. Also, some computational studies exist, such as those on LTA formation demonstrating the (theoretical) construction of D4R from dimeric aluminosilicates (Si(OH)_3_–O–Al(OH)_3_^−^) as a first step to LTA (Si/Al = 1).^145^ The fact that Al incorporates in certain ring structures (*e.g.* D4Rs) and avoids particular ones (5MRs) may be of large importance, provided that zeolites grow from the assembly of structural components (CBU, SBU, RBU) as commonly regarded, but difficult to prove experimentally. As mentioned earlier (3.1), the correlation of IZC parent–daughters *via* RBUs (with particular Al tendency) is striking,^40^ but this may be related to the chemistry of Al rather than due to its structural features. It is interesting to consider the proposed RBU approach by Suhendar *et al.*^40^ (and applied by Dos Santos)^127^ from a chemical (charge balancing) rather than from a structural perspective due to the particular role of Al in it.

Taken altogether, it is not (yet) possible to directly elucidate the nature and evolution of aluminosilicate precursors (*X*) taking part in (especially) nucleation and growth, also for those dissolving from crystalline lattices. The reactivity of ‘nanoparts’ is questionable as cyclic aluminosilicate oligomers are the more stable and less reactive species.^137^ The question remains whether this statement also holds in dense synthesis media and with symmetrical oligomers originating from crystalline dissolving units.

#### 3.2.4 IZC nucleation from a charge-balance perspective

**Fig. 10:**
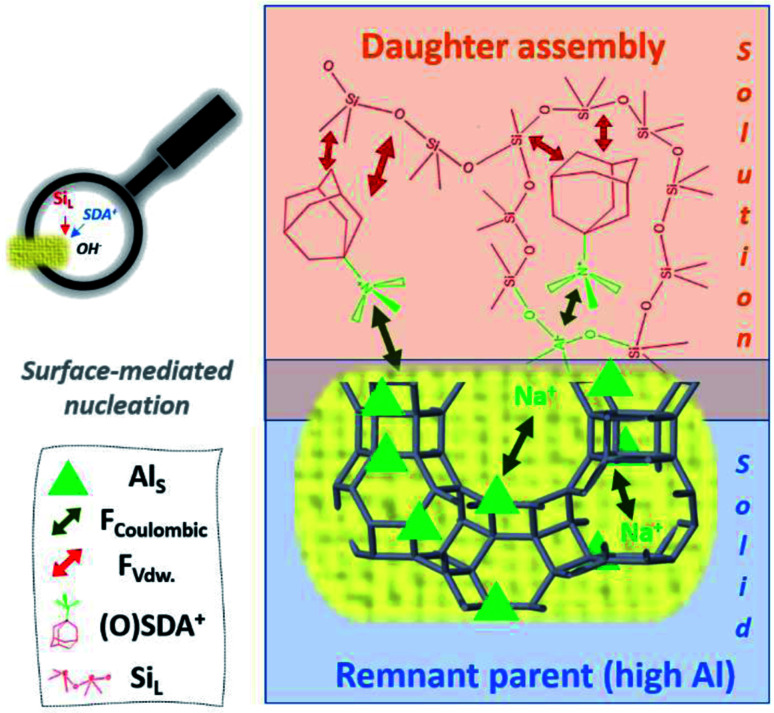
Representation of the hypothetic charge-balance model for generically fast IZC nucleation based on the findings of ref. 47 and linked to the IZC outcome after dissolution in Fig. 7. Swift heterogeneous nucleation is believed to take place at the surface of microporous and Al dense (parent) remnants due to the high ability to attract SDAs and dissolved T-atom species. Participating species and involved forces are labelled down left (resp. Al in the solid, coulombic forces, van der Waals forces (*F*_vdW_), structure-directing agents, and dissolved silicate species).

Above, we have re-interpreted the role of Al on (incongruent) dissolution (3.1) and on the subsequent nucleation mechanism (3.2.2). From these, it is very difficult to encapsulate the precise structures of the reactants participating in the chemistry of nucleus formation (3.2.3), let alone know the role of Al. Alternatively, the crucial role of Al in IZC nucleation was recently proposed from a charge-balancing perspective, considering the unique physicochemical properties present at the onset of nucleation (3.1.3). This allows an intuitive explanation for generically fast IZC nucleation as depicted in Fig. 10, in line with the recent findings of our group.^47^ During incongruent dissolution, an aluminium rich remnant persists with a large surface area (Fig. 5, 3.1.3), as it maintains the parent crystallinity in part. This yields a relatively high amount of Al available to attract SDAs. Independent to the possible occurrence of a structural similarity enhanced nucleation process (5), the presence of a high concentration of SDA near to a surface and the concomitant high concentration of dissolved species in the (nearby) solution then yield a preferred context for (generically) fast nucleation observed in (true) IZC synthesis (Fig. 8). A high attraction of SDA to Al-rich remnants is not presumed to exclusively occur with smaller alkali SDA molecules (with high charge density and easily accessing encaged Al), but also for OSDA molecules, with their phase-transport properties.^101^ Zones *et al.* recently demonstrated OSDA exchange on (parent) zeolites proportional to the Al content at room temperature and also acknowledges its role at relevant IZC conditions.^53^

The availability of a unique physicochemical context (from incongruent dissolution of crystalline sources) in IZC synthesis mixtures and a focus on the charge-balancing role of Al (and other charged species such as SDA) thus provides a new theory to explain inherently fast nucleation taking place in IZC mixtures. The commonly endorsed theory of structural elements (‘nanoparts’) facilitating nucleation and growth is challenged by new findings on swift IZC between structurally non-resembling parent–daughters.^3,5,127^ As such, this new proposal may better explain why particular IZC crystallizations occur fast and selectively by taking into account more chemical (charge-balancing) arguments and not just structural considerations. Moreover, the theory of charge-balancing (on the level of zeolite cages and channels) explains the odd assembly behaviours obtained in FAU-to-MFI crystallizations better than from a structural perspective (see below).

### 3.3 Role of Al during growth

Whilst nucleation occurs seemingly fast in any IZC case (generic), a notable exception is found during assembly, highlighting the charge-balancing perspective on growth with the crucial role of Al therein (3.3.1). Below it is explained why charge-balancing may fulfil another role during growth than its role in nucleus formation. For this, temporal investigations on the role of Al throughout IZC are important.

#### 3.3.1 IZC assembly from a charge-balancing perspective

Crystal propagation (growth) requires a much lower degree of supersaturation than nucleation.^111^ This explains the auto-catalytic nature of zeolite assembly. During assembly, the same chemistry takes place as at the onset of nucleation. However, the chemical environment changes constantly due to gradients in reactants and produced species. T-atom condensations occurring during assembly release OH^−^ and (some) SDA species (salting out),^50,51^ which in their terms increase supersaturation in proximity to the assembling phase. This translates into sigmoidal shaped yield and crystallization curves in most (IZC) synthesis, only decelerating due to the lack of nutrients at the end of crystallization. Nevertheless, given the dynamic evolutions taking place during synthesis, the physical (viscosity) and chemical environments (Si/Al) may alter over the course of evolution in such a way that supersaturation drops completely with assembly (a lack of precursor species *X* at the assembly location), even despite overcoming the earlier higher supersaturation barrier.

**Fig. 11:**
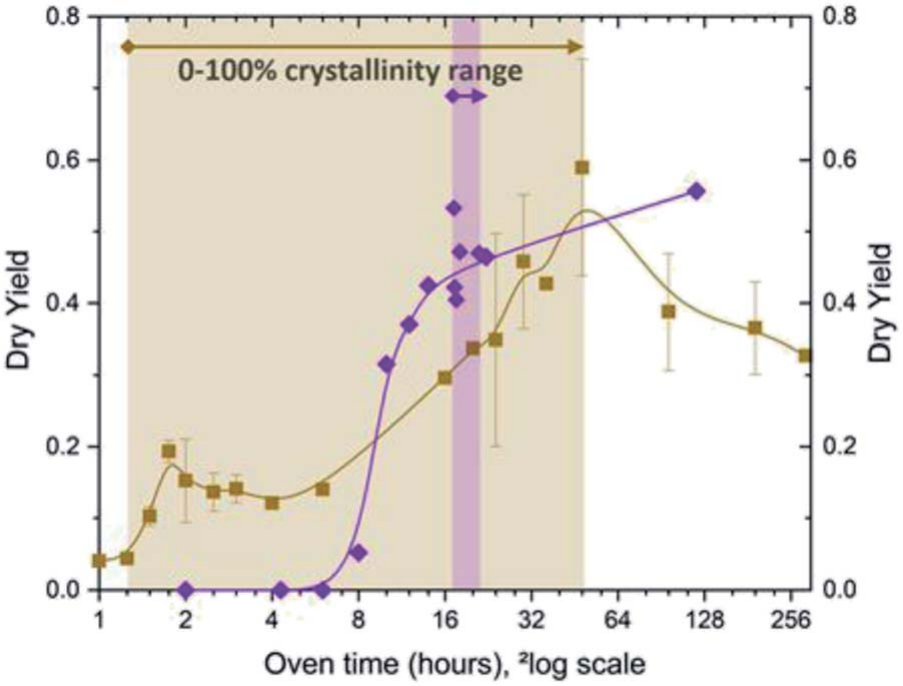
Temporal evolution of alkali-free ZSM-5 (MFI; Si/Al = 40) synthesis from amorphous sources (purple) and IZC (brown): dry yields (*y*-axis) and crystallinity ranges. The filled background represents the assembly stage (III) for each. Based on supporting info in ref. 47.

It is presumed that such an abrupt halt in assembly is observed in the high silicate FAU-to-MFI (Si/Al_P_ = 40, OH/Si = 0.35; no Na^+^).^47^ The alkali-free IZC nucleation occurred fast with X-ray visible ZSM-5 at 75 minutes of hydrothermal treatment in a context of Al rich sols (structure visualized in Fig. 7). However, growth decelerates immediately and stagnates 30 minutes later (solid yields in Fig. 11). Al content slowly drops from 0.35, the Al fraction reached earlier at equilibrium dissolution (see above, Fig. 4), to around 0.2 a few hours later. We suggest that this selective Al drop and growth stagnation occurs due to the inability of growing mixture to assemble Al. Hence, salting out of non-assembled Al during assembly of Si-rich fractions may hamper further growth as the propagating regions becomes even more Al rich. The mixture is presumed to lack the ability to charge-balance Al due to the intrinsic Si rich preference of the MFI topology^43^ and the nature and siting of SDA molecules. In this particular case, the exclusive presence of TPA promotes assembly of T-atom species with low charge density (mainly Si) *via* dispersive stabilization. Adding an alkali such as Na^+^ as co-SDA would add a high charge-density element allowing improved (coulombic) charge balancing of Al and subsequent swift assembly. A compositionally comparable IZC counterpart (FAU-to-MFI IZC; Si/Al_P_ = 40; OH/Si = 0.35) with identical overall charge density (OSDA^+^ + Na^+^/Al^−^) proved the latter.^47^ Fast assembly was demonstrated with a typical sigmoidal curve reaching complete crystallization within 2 to 4 hours. Sigmodal behaviour was also found in FAU-to-CHA IZC counterparts with and -importantly- without sodium (FAU-to-CHA; Si/Al_P_ = 40; OH/Si = 0.35). From the combined findings of these four kinetically studied IZC systems, a kinetic growth model for IZC is proposed with some generic features.^47^ In the latter it was motivated that IZC synthesis mixtures prosper particularly well in assembly conditions with preferential Al uptake (Al-loving), rather than in Al-averse ones (rather scarce).

After longer synthesis times, the peculiar Al-averse system (FAU-to-MFI, Si/Al_P_ = 40; OH/Si = 0.35; no Na) reaches a maximum (100%) crystallinity and yield (48 h) (Fig. 11, brown). Surprisingly, this is twice slower than its non-IZC counterpart from amorphous/soluble sources (see Fig. 11, purple).^47^ A gel-containing phase was encountered in both the IZC and the non-IZC syntheses before successfully achieving MFI (Si/Al = 40, alkali-free) crystals, seemingly indicating that a (slower) gel-mediated mechanism is necessary to assemble MFI with a relatively high content of Al in alkali-free mixtures. The later findings on Al-averse systems (alkali-free MFI) add additional arguments to the theory that different mechanisms prevail at different Al contents,^123^ and support growth theories in IZC mixtures based on charge-balancing.

#### 3.3.2 Influence of charge balancing on the extent of crystallization

To the best of our knowledge, this above-mentioned study is the first to report non-sigmoidal crystallization behaviour in IZC synthesis systems (apart from those with competitive crystallizations). Such findings were only made possible due to temporal follow-up of synthesis starting from relevant (dissolving) IZC conditions. An interesting evolution in this regard is the increased number of recent papers reporting temporal evolution in IZC mixtures, albeit most papers are limited to electron microscopy and (relative) crystallinity trajectories.^64,127,141–143,146–149^ It is not surprising that only sigmoidal shaped curves were found in all of these, as most syntheses investigate alkali-containing mixtures with high charge density (high SDA and Al concentrations), hence keen to assemble Al. Some interesting studies are those reporting (changes in) element yields throughout the crystallization process.^47,65,93,107^ Nishitoba *et al.* present exemplary temporal results in their LTL-to-CHA and FAU-to-CHA IZC (Si/Al_P_ = 3.0; Si/Al_B_ = 15, Fig. 6).^107^ Simultaneous LTL depletion and CHA growth occurs with decreasing K (salting out) and increasing OSDA contents in the solid respectively. As described earlier in 3.1.2, nucleation and growth occur at low Si/Al and low solid yields and both parameters increase with completion of crystallization. The final obtained yield of the systems is much lower in the TEA-containing system than in the TMAda-containing systems, probability due to the better (charge?) stabilization achieved with the larger (and tighter fitting) OSDA.^44^ In the end, all the investigated systems achieve an Al yield close to 100%, a common observation in IZC. This is also reflected by the extremely high Si/Al values reported in the liquid phase of completed IZCs.^54,65,93^ The latter is in part caused by incongruent dissolution of Si and Al at high alkalinity (see 3.1.1).

In most IZC investigations, tetrahedral Al is the only major coordination form detected by SS ^27^Al NMR throughout synthesis investigations, also in the encountered X-ray amorphous intermediates.^144^ Supporting the charge-balancing theories outlined above, it is proposed that the total yield will depend on the possibility to assemble SDA-framework composites that (charge) stabilize T-atoms. According to the latter, the lower (charge) affinity of Si, and its higher solubility at high pH will put a large limitation on Si yields. In case of a charge mismatch, this may lead to incomplete assembly and very low yields. The latter is often encountered in OSDA-free (seeded) syntheses^150^ or at border conditions of difficult to crystallize systems (*e.g.* AEI from a non-IZC^36^). Dusselier *et al.* has identified such systems (*e.g.* AEI and LEV) based on output Si/Al and SDA charge counts.^1^ Note that these crystallization systems may experience the same bottlenecks as the above described FAU-to-MFI, lacking further tendency (supersaturation) to assemble at the given (left-over) conditions in the liquid phase. For example, OSDA-free FAU-to-CHA only forms low Si/Al zeolites. Using higher Si/Al parent sources (Si/Al_P_ = 15) will achieve fast IZC kinetics (as apparent from particle morphology), but only gives a very low Si/Al (2.3) and concomitant low yields. A similar Si/Al (1.9) and much higher yields can be obtained from FAU-to-CHA with Si/Al_P_ = 2.6.^151^ Clearly, the available concentration of Si (multiple times higher) in the case of the Si/Al_P_ = 15 parent is only a minor factor in determining the extend of assembly, as charge-stabilization of Si/Al > 2 of CHA is not very feasible at the selected conditions (without OSDA). In well crystallizing systems at higher Si/Al, often containing OSDAs and providing lower density charge-balancing, solid yields can also be limited due to the high pH. In these, it is possible to augment solid yields by decreasing OH/Si ratios, although lowering this value too much will hamper parent dissolution, and eventually not lead to transformation. Instead, an alternative strategy is to lower the water content. In more dense mixtures, the silicate solute will be more condensed, and therefore leave a smaller fraction in solution.

### 3.4 Maturation

A simplistic view on crystallization dictates that synthesis will stop assembling at a particular equilibrium (*X* = X*; no supersaturation). This point is typically characterized by a maximum crystallinity (100%) reached and stable solid yields with prolonged hydrothermal treatment.^47,93,107^ The ensuing period of prolonged hydrothermal treatment is defined here as maturation (stage IV, Fig. 2). Next to the possible nucleation of a more (next) metastable phase, it is believed that zeolites may still be subjected to (internal) change as zeolites crystallizations are reversible by nature.^48^ The reversible nature of T–O–T condensation was experimentally detected by isotope labelling (^17^O), even in aqueous conditions at room temperature.^152^ Given the kinetic nature of assembly and the thermodynamic drive with prolonged hydrothermal treatment at elevated temperatures it is thus very likely that internal rearrangements occur (*cfr.* mechanism (1) in 3.2.2 (ref. 106)). Gallium mobility *via* intra-framework migration,^153^ Si island forming in silicoaluminophosphates,^154^ reorganization of Zr in Si-BEA ([Zr]-BEA formation),^155^ and Al mobility in multiple works on Al zoning^156–158^ are all literature examples of internal mobility by rearrangement, influenced by prolonged synthesis, all with practical implications for catalysis. The latter is not different in IZC synthesized zeolites. We proposed that internal rearrangements occur between framework incorporated T-atoms, based on decreasing divalent cation capacities (DCC) at prolonged hydrothermal treatment times.^47,65^ According to this, the (thermodynamic) driving force is charge-separation (from Al close to each other, to isolated Al) in line with the practical Dempsey's^113^ and Lowenstein's rules and charge-balancing theories (see above).

Sodium has a (charge) stabilizing role in zeolites, and thereby hampers framework mobility and yield variations in sodium-containing systems.^47^ Stable yields during maturation are also more likely seen in the presence of sodium in most literature investigations. However, very few alkali-free systems are considered in time. The yield of alkali-free ZSM-5 system drops significantly during maturation (see Fig. 11, brown).^47,65^ The latter may be linked to the lower stability of (incorporated) colloidal silicates in this Al-averse system. In contrast, the Al-loving sodium-free FAU-to-CHA (Si/Al_P_ = 40; alkali-free) synthesis has demonstrated slowly increasing (Si) yields with prolonged maturation.^65^ Similar yield evolutions were also detected in the study of Umeda *et al.* on alkali-free CHA and BEA synthesis made from identical batch compositions.^159^ The (Al-loving) CHA system showed increasing yields (from ∼50% to ∼70%), whilst the slower crystallizing BEA showed decreasing yields during maturation (from ∼55% to 35%). Presuming maturation directs (imperfect) zeolites towards better charge-stabilization and higher overall stability, it allows Al-‘loving’ frameworks to incorporate more assembling species X (silicates). The latter is probably not possible in Al-averse systems, as the excreted (non-incorporated) Al will most likely bind to dissolved (poly)silicates in solution. Note that this deduction of yield behaviour from a charge mobility perspective suits the view that charge-balancing (and the role Al takes in it) plays a crucial role in all aspects of zeolite assembly. It is also in line with the experimental evidence that is provided by the indirect DCC method but lacks direct evidence which can only be reached by advanced *in situ* methods, if even possible.^47^ Constant change in solution chemistry (and at the solid–liquid interphase) is also evidenced by eventual consecutive growth of competitive phases at prolonged maturation times (Ostwald rule of stages), for example in the formation of ANA after GIS-to-LEV^101^ or the formation of MOR (low Si) and quartz (high Si) after FAU-to-MFI.^41^ This last consecutive transformation into a more stable siliceous phase (quartz) and a more aluminous MOR experimentally showcases the thermodynamically driven nature of selective formation of phases with a particular Al tendency. The latter, once more, highlights the important role of local Al charge-balancing.

## 4. IZC using other heteroatoms

The synthesis and commercialization of stannosilicates (*e.g.* TS-1, ([Ti]MFI))^160^ and silicoaluminophospates (*e.g.* SAPO-34 (CHA))^26^ for selective oxidation catalysts and methanol to olefins (MTO) respectively, has expanded the field of zeolites to contain heteroatoms other than Al (zeotypes). The hydrophobicity and the (Lewis-)acidic nature of the heteroatom containing zeotypes make these materials promising candidates in numerous redox catalysis reactions.^161–170^ Alongside direct (*in situ*) synthesis, these materials can also be formed post-synthetically.^171–174^ Typically this encompasses a two-step method involving chemical leaching and (heteroatom) impregnation.^170^ However, the impregnation (often aqueous exchange) is often difficult to achieve due to competitive H^+^ exchange at low pH or precipitation at high pH.^175^ Along with low intra-framework mobility of larger elements (*e.g.* Sn or Ti), post-synthetically produced catalysts also suffer from reduced hydrophobicity due to the difficultly of healing the Si nests formed.^176–184^

Many hydrothermal synthesis routes involve the use of fluoride to obtain hydrophobic zeotypes.^169,170^ Fluoride is environmentally unfriendly and hazardous, but often essential to obtain certain zeotypes. In (fluoride-free) alkaline media however, many zeotype synthesis routes suffer from inferior crystallization behaviour, especially compared to their (alumino)silicate versions (see below). Therefore, it seems opportune to apply the IZC strategy for other zeotypes as well, given the ease of transformation. In this regard, we review the scarce works on ‘interzeotype conversions’ and try to link it to the mechanistic interpretation for Al made above, where possible.

Table 1 reports the complete set of ‘true’ IZCs found in open literature (only 6 entries), supplemented with some ‘partial’ IZC forms, either with the metal included in the parent or with the heteroatom sources added in solution. Zones pioneered the field and reported the use of calcined boron-beta zeolite as the source of both boron and silicon in the formation of [B]CON (SSZ-33) as early as 1994.^185^ More recently, IZC synthesis of zeolites containing Sn,^186–188^ Fe,^188–190^ Ga^188^ and Ti^191–193^ have been reported as well (Table 1). Additionally, SAPO and ALPO IZC have been reported and a novel SAPO framework has been discovered (RHO, entry 4) using this synthesis technique.^194–196^

**Table 1:** IZC of heteroatom containing zeolites

Entry	Parent^a^	Si/M^*x*+^ in batch	Seeds (wt%)	Daughter^a^	Ref.
**Full IZC**					
1	[B]BEA	4–10	—	[B]AFI	185
2	[Ti]MWW**	53	Si-BEA* (10)	[Ti]BEA	191
3	[Al;P;Si]AFI (SAPO-5)	N.A.	—	[Al;P;Si]CHA (SAPO-34)	194
4	[Al;P;Si]AFI	N.A.	—	[Al;P;Si]RHO (DNL-6)	194
5	[Al;P;Si]FAU (SAPO-37)	N.A.	—	[Al;P;Si]LTL	195
6	[Al;P]AFI (ALPO-5)	N.A.	—	GAM-2^+^ ([Al;P]XXX)^d^	196

**Partial IZC**					
7	[B]BEA	16.5^c^	—	[B]CON	185
8^b^	MWW**	63^c^	Si-BEA* (10)	[Sn]BEA	186
9	FAU*	80^c^	Si-BEA* (10)	[Sn]BEA	187
10	[Ti;Al]FAU*	24	—	[Ti;Al]AEI	192
11	[Ti,Al]FAU*	14–64	Al-CHA (3)	[Ti;Al]CHA	193
12	[Fe;Al]FAU*	97–380	—	[Fe;Al]CHA	188
13	[Ga;Al]FAU*	82–618	—	[Ga;Al]CHA	188
14	[Sn;Al]FAU*	29	—	[Sn;Al]CHA	188
15	[Al]FAU*	100^c^	—	[Fe;Al]AEI	189
16	[Al]FAU*	100^c^	—	[Fe;Al]ERI	190
17	[Al]FAU*	100^c^	—	[Fe;Al]AFX	190

a IZA 3-letter codes are used instead of material names.

b Fluoride based.

c Metal added to solution.

d GAM-3 (after calcination), no IZA code yet.; * de-aluminated; ** de-boronated; N.A. not applicable.

Zones and Nakagawa achieved fast nucleation and growth of borosilicates using boron-beta ([B]BEA) as a starting reagent in the presence of a variety of organic SDAs (Table 1, entry 1).^185,197^ Using other reagents ([B]MFI or amorphous sources), no reactivity was observed. Phase selectivity was also found to be dependent on the OSDA and concentration of borate in the reaction mixture. For example, adding additional B_4_O_7_ to the synthesis mixture shifted the phase selectivity from [B]AFI (B-SSZ-24) to [B]CON (B-SSZ-33) (Table 1, entry 7). The authors suggest that the presence of extra borate shifts the reaction equilibrium towards species which are needed for [B]CON, which itself requires higher amounts of lattice substitution (lower Si/B) to form. A parallel can be drawn to the phase selectivity relationships encountered in various OSDA containing Al-zeolite systems. For example, TMAda-crystallising systems dependent on the Al contents (CHA, STT and AFI formed at low to high Al contents respectively).^198^ This is likely related to the similar chemistry of B and Al in aqueous solutions. Both are weak acids, with boric acid being more acidic than (Al(OH)_3_).^199^

[Sn]BEA has recently become a catalyst of great interest due to its Lewis acidic behaviour even in the presence of water, which has the potential for a variety of industrial applications.^161–168,200^ However, traditional synthesis routes allow only a limited Sn content as the addition of Sn inhibits the nucleation of the [Sn]BEA zeolite and such routes are often time-consuming.^164,201^ Substantial effort has been undertaken to optimise the synthesis of [Sn]BEA and maximise the Sn content. Techniques such as steam-assisted conversion and utilising a Sn–Si mixed oxide precursor were explored.^201,202^ Unfortunately, the [Sn]BEA produced contained impurities due to the constraints of the methods used. Contrastingly, the IZC strategy produced a pure [Sn]BEA with high Sn content. Zhu *et al.* reported the synthesis of pure [Sn]BEA through IZC from all-silica MWW with a high Sn content (Si/Sn = 63, 3.03 wt% Sn) in a reduced time frame of 3 days in fluoride containing media (Table 1).^186^ The [Sn]BEA from IZC had reduced crystal size, comparable hydrophobicity and, in their comparison, a superior catalytic performance.^186^ The higher Sn content also benefited product selectivity highlighting the promise of IZC strategies. More recently, Zhu *et al.* reported the synthesis of [Sn]BEA *via* the IZC of USY without the aid of fluoride and alkali metals.^187^ The Sn-BEA synthesised was reported to have the smallest crystal size (50–150 nm) among present synthesis strategies, which was said to result in good diffusion properties and relieved steric restrictions, which resulted in high activity for the Baeyer–Villiger oxidation of ketones.

The same group that developed the [Sn]BEA IZC methods were able to synthesise [Ti]BEA from [Ti]MWW in a solvent-free system within 2 h. The latter may be a ‘true’ IZC case (Table 1, entry 2), although the high content of seeds should be noted.^191^ Their nanosized [Ti]BEA produced showed high catalytic performance in the oxidation of cyclohexene with aqueous H_2_O_2_, likely due to the increased hydrothermal stability of the Ti^4+^ species and smaller crystal sizes, as suggested by the authors. It should be noted that all 3 works of the Zhu group use 10 wt% of dealuminated beta as seeds.^186,187,191^ These are necessary to accelerate the synthesis, or even for phase selectivity, indicating that IZC synthesis conditions were not ideal in these works. Partial IZC has been used to form Fe containing small pore zeolites in high yields although in examples shown in Table 1 (entries 10–17), Al was also present. These catalysts have shown promise for NH_3_-SCR of NO_*x*_ when combined with Cu.^188–190^

Zones and Nakagawa regarded source materials as the most determinant/critical factor in IZC,^185^ a conclusion similar as for Al-IZC. The latter may explain the start of IZC conversions from very high silica zeolites, or dealuminated zeolites (Table 1).^186,187,191^ The siliceous sources, present in highly alkaline synthesis mixtures, undergo fast dissolution and high silica solubilities leading to high source dissolution (see 3.1). In parallel with Al, other elements such as iron tend to condense in highly alkaline media more with silicates than to themselves.^80^ Due to this condensation tendency on silicate surfaces, the concentration of the heteroatoms (*e.g.* Fe) in solution will remain low, which may explain why (hydr)oxide forms (*e.g.* SnO_2_) are not typically encountered, despite the very high pH.^186^ Very high heteroatom contents have been reported to inhibit growth, especially in the case of partial IZC with external Sn.^164,186,187,201^ Likewise as for Al, precipitation against large (amorphous) silicates will hamper further dissolution, leading to lower supersaturation hampering crystallization (*cfr.*Fig. 7). Furthermore, high heteroatom content may influence aggregation of larger particles (gel formation), hence, reducing further the dissolution of silicates (OH^−^ based) and influencing saturation. Taken all together, seemingly similar processes take place accelerating and hindering dissolution and supersaturation as in conventional Al-IZC, described in 3.1.3. The tendency of M^*x*+^–O–Si bond formation is pinpointed as a factor in all of these reversible condensation–polymerization reactions. Properties such as bond-length, charge density and nucleophilicity may all contribute to this reaction.

Either way, IZC may be a good strategy to (partially) overcome the problem of reduced M^*x*+^ incorporation when M^*x*+^ is already present in the parent form. M^*x*+^ present in dissolving parent entities may protect its surroundings from dissolution, due to preferential Si–O–Si dissolution in alkaline media and hereby keeping M^*x*+^ incorporated. However, whether or not this is the case for Sn–O–Si, Zr–O–Si and other larger element adducts remains questionable.

Zhu *et al.* reported late Sn incorporation in the framework, with respect to crystallization and yields (Si incorporation).^186^ Perhaps, internal changes during maturation time (at high pH) allows a better siting of (larger) heteroatoms. The latter was also suggested from analysing the work of Kots *et al.* on [Zr]BEA synthesis.^155^ In this temporal zeotype synthesis study (which are scarce), they found crystallization occurring prior to Zr framework incorporation, taking place at constant solid yield and Si/Zr (a solid-mediated processes). Specifically, the Lewis acidity of the obtained materials was found to increase with prolonged hydrothermal treatment, suggesting progressive framework incorporation of Zr (in closed framework sites). The latter ‘maturation effect’ may be of importance for any type of zeolite, as suggested from the results of Zhu *et al.* on stannosilicates,^186^ and more general for Al-containing zeolites (see 3.4). It can be expected that the more flexible zeotypes (and severe hydrothermal conditions, *i.e.* high temperatures) allow the highest heteroatom incorporations. Though it should be mentioned that heteroatom incorporation is also largely influenced by the thermodynamic tendency to incorporate these heteroatoms (*cfr.* Al).^203^ Aspects such as bond-strengths, heteroatom solubilities in alkaline media and other contributors may influence synthesis to an unknown extent. In this context, it is currently not possible to capture the importance of charge-balancing effects (*e.g.* a neutral stannosilicate *vs.* a heavily charged zincosilicate).

In summary, IZC has shown promise for heteroatom-substituted zeolite materials with fast crystallisation times and nanosized crystals, and high degrees of metal incorporation. The key advantages of IZC (selective nucleation, shorter synthesis times, high yields) can be related to the success of heteroatom dissolution and incorporation. The key determinants are likely M^*x*+^–O–Si bond formation tendency in alkaline media and the resulting physical states present at the onset of nucleation, *cfr.* for Al-IZC (3.1.3, Fig. 7). IZC is predicted to achieve higher supersaturation, thus a more direct phase formation (*S*_T_ > *S*_K_, Fig. 8), achieving a higher chance for successful zeotype synthesis as compared to conventional sources. Furthermore, synthesis time (maturation) was found to be crucial for heteroatom incorporation in certain zeotypes (Zr or Sn-BEA). Combined with other successful zeotype synthesis methods, such as seeding^184,186,187,191^ IZC is predicted to further prosper the development of superior zeotype materials, a constantly evolving field with emerging applications.

## 5. General conclusions and future perspectives

IZC synthesis is, as other conventional syntheses, still an unravelled sequence of complex coupled dissolution–precipitation reactions with a lot of degrees of freedom. General thermodynamic indications (*e.g.* framework densities) and kinetic adducts (nanoparts) are not sufficient to predict particular parent–daughter relations. Given parent–daughter success related to ring building units (RBU, *e.g.* 5MR or 6MR) and the particular tendency for these rings to contain Al,^40^ a marriage between the role of Al and the nanoparts theory could be suggested. In this perspective, a chronological series of synthesis aspects were discussed: the dissolution behaviour of parent zeolites, the chemistry of the physical states present after dissolution, nucleation theory in zeolites and the influence of Al on assembly and maturation. From all of this, the key role of Al during dissolution and at the onset of nucleation, as well as the particular importance of charge-balancing for consecutive growth (assembly) was identified as crucial to IZC syntheses. This perspective contains some insights that can be sequestered from the analysis. The key take-aways are listed below per stage.

### 5.1 Stage I: dissolution

- Source dissolution can be controlled by a limited amount of DOFs (Fig. 3), more easily than *via* conventional sources involving separate Si and Al sources.

- Zeolite sources dissolve incongruently, due to the higher reactivity of hydroxides to silicates than to aluminates (Fig. 4).

- The remnant solid becomes denser in Al, in part due to reversible Al condensation.

- Relatively homogeneous elemental compositions are maintained in (Al-rich) remnants sols (Fig. 5), contrasting with some amorphous gel recipes, encompassing a passivation layer (Fig. 7).

- Zeolite dissolution is most often the rate limiting step.^54^

### 5.2 Stage II: induction and nucleation

- The physical states obtained at the onset of nucleation achieve either a higher supersaturation, or swift nucleation at low *S* (Fig. 8, right), or a combination of both, as evidenced by smaller and more daughter crystals.

- High supersaturation occurs most likely due to fast IZC dissolution, making more precursor species (*X*) available. High supersaturation enables more direct synthesis routes, rather than kinetic routes *via* amorphous gel phases. The frequently reported absence of gel-like phases may be a witness of nucleation at high supersaturation (*S*_T_).

- The aluminous (and large external) surface has a high tendency to attract SDAs,^53^ which allows concentration of (pre-)nuclei to form in proximity of the solid surface (Fig. 9).

- Nucleation occurs heterogeneously, either selectively (structurally enhanced) or non-selectively with an important role for the solution (as provider of precursor species).

- The precise chemical entities participating in growth are not known to date. Analysis of the chemistry suggests that (stable) cyclic components are not reactive enough to participate in nucleation and assembly.^137^

### 5.3 Stage III: growth

- Growth assembly occurs *via* the same reaction pathways as nucleation (condensation–polymerisations and stabilization mechanisms). However, the assembly chemistry (salting-out) changes the local chemical environment.

- From a chemical viewpoint, potential crystal assembly of oligomers most likely occurs *via* (reactive) linear, rather than *via* (stable) ring structures.^137^

- Particular preference for framework assembly of either Al or Si containing T-atoms depends on charge-balancing. Preference of Al (Al-loving systems) occurs at higher charge densities than Si assembly (Al-averse systems).

○ Selective Al assembly leads to quick (sigmoidal) IZC growth, as in most reported cases.

○ Al-averse assembly is halted in IZC conditions, despite very quick nucleation, in a particular crystallization system preferring Si assembly (ZSM-5 synthesis with only TPA^+^; Fig. 11).

### 5.4 Stage IV: maturation

- All synthesis mixtures keep evolving after prolonged synthesis time. Internal rearrangements of framework T-atom bonds (T–O–T) takes place, and are even possible at room temperature.^152^

- The latter allows charge-balancing if enough mobility is allowed by the flexibility of the framework, especially at higher synthesis temperatures as T–O–T condensations are a thermally activated process.

### 5.5 On heteroatom zeolites (chapter 4)

- Few published *interzeotype conversions* are found in open literature (Table 1). These show the large potential of the IZC strategy in term of synthesis and output performance (selective nucleation, shorter synthesis times, high yields).

- A large spectrum of elements can be incorporated in zeotypes *via* this technique: B, Fe, Ga, P, Sn and Ti.

- Extended synthesis times (maturation), can change the acidic nature and the connectivity of the heteroatoms, holding important consequences for catalysis.

- IZC success is likely related to the same factors as Al-IZC success. However, in-depth mechanistic investigations are lacking and many questions remain:

○ How is the M^*x*+^–O–Si *versus* Si–O–Si reactivity in alkaline media? We assume that most metal-silicate bonds (*e.g.* Fe–O–Si)^80^ are more resistant to dissolution than Si–O–Si, leading to a similar densification as with Al.

○ What are the resulting physical states present at the onset of nucleation? And the speciation of heteroatoms in solution?

○ How is the charge-balancing influenced? M^4+^ heteroatoms (Ti, Sn, *etc.*) and Zn^2+^ elements imply a significantly different charge imbalance?

### 5.6 Practical insights and future outlook

We have emphasised the role of Al in this bond-formation chemistry, and would like to underline its importance to achieve zeolites (or zeotypes) with desired topologies and acidities *via* IZC. Few external manipulations (*e.g.* temperature change, *etc.*) are performed during conventional batch synthesis over time. Hence, all the “intelligence” is present within the starting materials and the pre-selected conditions.^73^ Thus, the initial degrees of freedom (DOFs) influencing the first stage (dissolution, see 3.1) will be determinant for all consecutive reactions. Differences between nucleation in IZC mixtures *versus* those from conventional (amorphous) sources at similar compositions are entirely determined by the physical states present at the onset of nucleation, in their turn a consequence of source dissolution (see 3.1, Fig. 7). It is questionable whether general structural features (*e.g.* framework type, framework density, structural similarity) are determinant for dissolution kinetics. Instead, the role of Al and its consequence on the physical stages during or after dissolution should be considered and investigated in detail *via* comparative syntheses. Zeolites are known to be far from ideal systems. Hence, the role of structural defects (dealumination) may also be underestimated and it is highly recommended to characterize the parent materials. Often parent zeolites pre-treated by dealumination are used as source materials, without acknowledging the higher tendency of dissolution of these materials superior dissolution properties (higher supersaturation *S*_T_) can explain some IZC successes much better than the often claimed parent–daughter ‘structural similarity’.

Using commercial benchmark parents should be encouraged, including additional information and characterisation as differences between production batches may exist from time to time.

Successful IZC requires judicious selection of dissolution conditions, especially in systems with narrow crystallization ranges (*e.g.* AEI). OH/T-atom is a good parameter in this respect, as demonstrated recently (Fig. 9)^132^ and we suggest to further explore the OH/Al parameter in future IZC studies. This parameter contains the role of the incongruent dissolution and the nature of Al most directly.

The synthesis performance of a particular IZC system should be compared to a peer-system with nearly identical synthesis conditions from amorphous or soluble precursors (single-parameter variations).^47,204^ It is advised not to make changes in the overall charge balance, *e.g.* by working at similar pH and constant ionic strengths (SDA/Al).

In terms of synthesis performance, higher yields can be obtained at lower water contents. H_2_O-lean solutions achieve relatively higher supersaturation (*S*_T_, more direct formation routes) and have a higher concentration of silicates in solution, allowing a higher (relative) amount of silicates into the final (zeolite) product. This may also benefit framework selectivity, such as in the FAU-to-CHA using TEA, which is only possible at low water contents.^63^

Alkali-free syntheses should also be targeted, given the success in particular IZC trials (*e.g.*Table 1; entry 2).^191^ Increasing focus on the role of alkali (such as sodium) on dissolution and crystallization could further direct to beneficial IZC pathways.

It is also recommended to use IZC in combination with other synthesis strategies (see 2.2) to obtain materials with unprecedented properties, or, zeotypes. For one, the charge density mismatch (CDM)^205^ strategy should be considered in IZC syntheses. This has not yet been reported explicitly, but could exploit charge-balancing (and Al) consideration similar as presented here.

Some heteroatom elements have not yet been targeted using IZC (*e.g.* Zn), despite hydrothermal synthesis successes using conventional sources. Expanding efforts on ‘*interzeotype conversion*’ is expected to yield new materials (*i.e.* framework types), as well as useful materials in terms of application potential.

Kinetic (temporal) analysis of IZC syntheses are scarce, but very useful to obtain both practical and fundamental insights into IZC synthesis, especially taking into account Al contents in both liquid and solid as demonstrated in this Perspective. We envisage that temporal analysis of IZC intermediates will become crucial to achieve increased understanding of the processes determining IZC success. Some of the characterization strategies that could be considered are the following:

- Assessment of pH of the mother liquids, as well as morphology (defects) and surface chemistry of the zeolitic parents prior to high temperature reactions.

- A focus on the evolution of Si/Al during temporal IZC tracking, rather than on liquid/solid yields, as the latter are rather determined by colloidal phenomena (aggregation) and separation (centrifugation). The former contains more valuable information regarding connectivity and heterogeneity of the precursor sources, especially combined with an analysis on the acid site distributions.^47^

- In-depth investigations of the intermediate stages, revealing information on both the physical states present as well as the detailed chemical entities (acid sites?) using a broad selection of techniques: TEM with elemental mapping, spectroscopic methods (FT-IR, Raman), diffraction methods (SAXS, XANES, …).

- Ideally, *in situ* characterization should be envisaged, such as done for conventional syntheses.^50,51^*Ex situ* tests may be biased from (oven) cooling (*e.g.* precipitation) and necessary treatments executed on the samples (*e.g.* calcination).

- Investigations into the nature of chemical species (oligomers) involved in crystallization should be envisaged.^4^ If not allowed by current technologies, computational studies could help to further uncover the role of dissolution thermodynamics.^138^ In this regard, only indirect measurements exist. Interesting to mention is the very recent effort of Dedecek *et al.*, who claimed to have traced the accumulation of ‘linear’, rather than cyclic, components participating in IZC assembly, from their solid state (SS) NMR study.^206^

- Characterization of the as-made materials should be regarded prior to analysis of calcined versions, since calcination alters zeolitic properties (defects, …). In this light, differential TGA can reveal part of the charge-balancing information of the formed as-made materials. Also, SS NMR ^27^Al and ^29^Si NMR on non-calcined solids is welcome, especially, on samples present at the onset of nucleation.

## Author contributions

J. D. wrote the original draft. M. S. mainly contributed to the heteroatom zeolite section (Section 4). M. D. supervised the writing and all authors actively conceptualized and edited the whole manuscript.

## Conflicts of interest

There are no conflicts to declare.

## Supplementary Material
